# Dual Catalytic Activity of a Cytochrome P450 Controls Bifurcation at a Metabolic Branch Point of Alkaloid Biosynthesis in *Rauwolfia serpentina*

**DOI:** 10.1002/anie.201705010

**Published:** 2017-07-12

**Authors:** Thu‐Thuy T. Dang, Jakob Franke, Evangelos Tatsis, Sarah E. O'Connor

**Affiliations:** ^1^ Department of Biological Chemistry John Innes Centre Colney Lane Norwich UK

**Keywords:** alkaloids, biosynthesis, cytochrome p450s, perakine, vomilenine

## Abstract

Plants create tremendous chemical diversity from a single biosynthetic intermediate. In plant‐derived ajmalan alkaloid pathways, the biosynthetic intermediate vomilenine can be transformed into the anti‐arrhythmic compound ajmaline, or alternatively, can isomerize to form perakine, an alkaloid with a structurally distinct scaffold. Here we report the discovery and characterization of vinorine hydroxylase, a cytochrome P450 enzyme that hydroxylates vinorine to form vomilenine, which was found to exist as a mixture of rapidly interconverting epimers. Surprisingly, this cytochrome P450 also catalyzes the non‐oxidative isomerization of the ajmaline precursor vomilenine to perakine. This unusual dual catalytic activity of vinorine hydroxylase thereby provides a control mechanism for the bifurcation of these alkaloid pathway branches. This discovery highlights the unusual catalytic functionality that has evolved in plant pathways.

The plant *Rauwolfia serpentina* (Apocynaceae), commonly known as Indian snakeroot, has traditionally been used for medicinal purposes in South and South‐East Asia for its antihypertensive and calming effects. This plant is also one of the fifty fundamental herbs in traditional Chinese medicine.^1^ *Rauwolfia* spp. produce a wide variety of compounds, including approximately 150 monoterpene indole alkaloids (MIAs) such as reserpine, yohimbine, and raubasine.^2^ One of the best known MIAs of *Rauwolfia* is the ajmalan‐type MIA ajmaline, a class Ia antiarrhythmic agent often used in diagnosis of patients suspected of having Brugada syndrome.^3^ Like all MIAs, ajmaline is produced via the versatile intermediate strictosidine, which is subject to multiple catalytic steps to yield the structurally diverse members of the MIA natural product family.^2^ All biosynthetic transformations leading from strictosidine to ajmaline have been detected in enzyme fractions from *Rauwolfia* cell culture, though only six biosynthetic genes have been cloned and characterized (Figure 1).^4^, ^5^, ^6^, ^7^, ^8^, ^9^, ^10^, ^11^ Several studies showed that vomilenine is an ajmaline biosynthetic intermediate that is produced through selective hydroxylation of vinorine at the C‐21 position by *Rauwolfia* cell culture extracts (Figure 1).^12^, ^13^, ^14^ However, no enzyme isolated from *R. serpentina* has shown this activity.^11^

**Figure 1:**
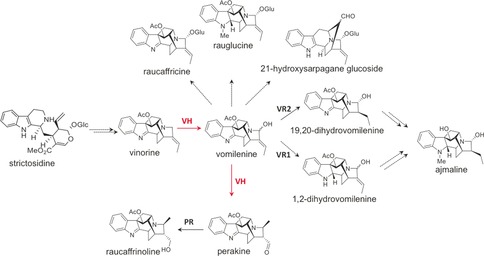
Alkaloid network in *R. serpentina* from strictosidine. Vomilenine is the central intermediate for the metabolic pathway to ajmaline, raucaffrinoline, raucaffricine, and 21‐hydroxysarpagane glucoside. Two routes from vomilenine to ajmaline are proposed. Functionally characterized enzymes are noted in black. Broken arrows represent uncharacterized reactions. The enzyme from this study is shown in red. VH=vinorine hydroxylase, VR1=19,20‐vomilenine reductase, VR2=1,2‐vomilenine reductase, PR=perakine reductase.

Comparative transcriptomics and metabolomics have been widely and successfully used to elucidate the genes of plant alkaloid metabolism.^15^, ^16^, ^17^, ^18^ Here we report the use of an available *Rauwolfia* transcriptome (Medicinal Plant Genomics Resource (MPGR), http://medicinalplantgenomics.msu.edu/index.shtml) to identify and characterize a cytochrome P450 enzyme (CYP82S18) that hydroxylates vinorine to generate vomilenine, which we show exists as rapidly interconverting epimers in solution. Surprisingly, this cytochrome P450 also catalyzes the redox neutral isomerization of vomilenine to form the alkaloid perakine. Therefore, this second, non‐oxidative function of the cytochrome P450 provides an unexpected route to the diversification of the ajmalan alkaloid scaffold.

Early studies using *R. serpentina* protein extracts suggested the involvement of a cytochrome P450 (CYP) in the conversion of vinorine into vomilenine.^19^ The expression profiles of genes encoding CYPs from an available *Rauwolfia* transcriptome database were used to search for candidates.^20^ Approximately 270 transcripts are annotated as cytochrome P450s in the *R. serpentina* transcriptome. Of these, 110 transcripts are expressed in young and mature root tissues, where ajmaline is highly accumulated. Transcripts from the dataset were clustered using a self‐organizing map (SOM; Figure S1 in the Supporting Information) for efficient candidate identification.^21^ The search for vinorine hydroxylase focused on 1) candidates with gene expression profiles correlated with the accumulation of ajmaline and 2) candidates within or adjacent to nodes containing previously identified MIA enzymes. Available metabolomic data suggested that while ajmaline is found in the highest levels in young roots, aerial organs display trace to undetectable ajmaline levels (Figure 2 A). Correspondingly, transcripts encoding known enzymes in the ajmaline pathway were most highly expressed in roots (Figure 2 B). Eight CYP candidates from different families (Figure 2 C) that displayed similar expression profiles to known ajmaline genes were identified from analysis of the SOM.

**Figure 2:**
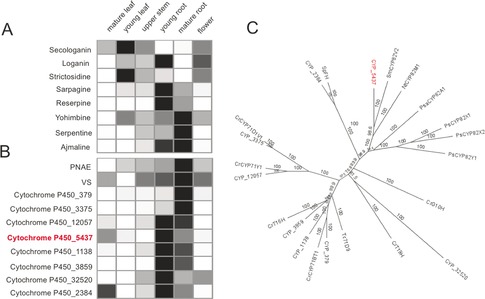
Heat maps showing the relative abundance of major alkaloids (A) and transcripts encoding selected known enzymes and CYP candidates (B) in different organs of *R. serpentina*. C) Unrooted neighbor‐joining phylogenetic tree for CYP candidates from this study and other reported CYPs from other organisms. Bootstrap frequencies for each clade were based on 1000 iterations. Abbreviations and GenBank accession numbers for each protein are provided in the Supporting Information.

The coding regions of the CYP candidates were inserted into the multiple cloning site of a yeast dual‐expression vector with the required cytochrome P450 reductase CPR. To determine the enzyme activity of CYP candidates, 10 μm of the substrate, vinorine, was fed to 1 mL yeast cultures for 48 h. Only yeast cultures that harbored the construct encoding candidate 5437 (pESC‐leu2d:CPR/5437) showed the consumption of vinorine and the formation of two new products (Figure 3 A). No enzymatic product was observed when vinorine was incubated with yeast cultures harboring empty vector, or any of the other CYP candidates. Similarly, in vitro assays with microsomal fractions of yeast harboring pESC‐leu2d:CPR/5437 also showed that in the presence of NADPH, vinorine (*m*/*z* 335) was consumed by the enzyme, resulting in the formation of two reaction products with *m*/*z* 351 as evidenced by LC–MS analysis (Figure 3 A). In vitro assays with all other candidates did not show any consumption of vinorine. The substrate specificity of 5437 was determined using 10 MIAs representing several different structural subgroups (Figure S2). Vomilenine, the hydroxylated product of vinorine, was the only other alkaloid accepted by 5437, albeit with much lower efficiency (13 % of the substrate turnover rate compared with vinorine; Figure 3 B). This high substrate specificity is consistent with previous work with microsomal extracts of *Rauwolfia*.^14^ The closest homologue of 5437 in the related Apocynaceae plant *Catharanthus roseus* (cra_locus_12789) did not accept vinorine as a substrate.

**Figure 3:**
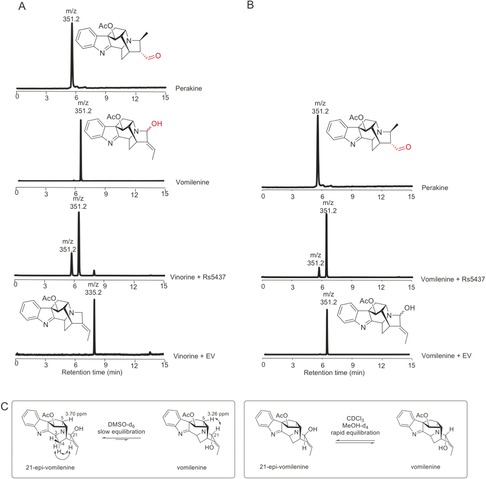
Catalytic function of recombinant cytochrome P450_5437 candidate (5437). A, B) Extracted ion chromatograms showing the in vitro catalytic activity of 5437 (CPR/5437) using vinorine (A) and vomilenine (B) as substrate, compared with the negative control (CPR). C) Only the microsomal preparations containing recombinant 5437 with vinorine (*m*/*z* 335) yield two reactions products. Vomilenine occurs as C‐21 epimers in solution. CPR=cytochrome P450 reductase.

The major enzymatic product of 5437 had a mass of *m*/*z* 351, an increase of 16 amu compared to the vinorine starting material, which strongly suggests that 5437 catalyzes a hydroxylation reaction. A minor enzymatic product with *m*/*z* 351, but a different retention time, was also observed. To identify the reaction products, a large‐scale reaction was carried out, and the major product was characterized by NMR analysis (COSY, NOESY, HMBC, HSQC). Both ^1^H and ^13^C chemical shifts were in good agreement with previously reported data for vomilenine (Figure 1, Figure S6, Table S1).^13^, ^22^ 5437 was therefore designated vinorine hydroxylase (VH). This enzyme displays an optimal pH of 6.5 for the hydroxylation reaction (Figure S5), which is lower than previously reported for the reaction catalyzed by *R. serpentina* microsomes.^19^ The optimal pH for the isomerization reaction, which was not reported to be associated with VH activity in *R. serpentina* microsomes,^19^ was 4.5 (Figure S5). Under optimal conditions (HEPES‐buffered reaction at pH 6.5 and 30 °C; Figure S5), recombinant VH followed Michaelis–Menten‐type reaction kinetics with an estimated *V*_max_ value of 98 pmol min^−1^ mg^−1^ total protein and a *K*_m_ for vinorine of 6.8 μm (compared to a *K*_m_ of 23 μm *R. serpentina* microsomes^19^; Figure S5). No significant substrate or product inhibition was detected.

The stereochemistry of the newly installed hydroxy group at C‐21 of vomilenine was assumed to have the (*R*) configuration, based on the stereochemistry of the downstream product ajmaline. However, careful NOESY analysis suggested that the vomilenine product exists as a mixture of epimers at C‐21, based on the co‐existence of NOE contacts between H‐21 and H‐3, H‐14, and H‐5 in CDCl_3_ and [D_4_]MeOH (Figure 3 C, Figure S8). In both solvents, only a single signal set was observed, thus indicating a rapid equilibration on an NMR timescale in these solvents. In contrast, two signal sets in a ratio of approximately 4:1 were observed in [D_6_]DMSO. Based on NOE data, these signal sets could be clearly assigned to the C‐21 epimers, with 21‐epi‐vomilenine being the main component (Figure S8). This assignment is also supported by the strong deshielding of H‐5 of 21‐epi‐vomilenine (δ_H_=3.70 ppm), caused by the proximity of the C‐21 hydroxy group, in comparison to vomilenine (δ_H_=3.26 ppm; Table S1). A previous report showed that this slow equilibration can be accelerated by addition of water,^23^ thus suggesting that the epimerization could occur under physiologically relevant aqueous conditions. Given that plant CYPs in specialized metabolism typically catalyze highly specific reactions, it is most likely that VH catalyzes a regio‐ and stereoselective hydroxylation reaction that is followed by isomerization, although oxidation by VH through hydride abstraction to form the iminium moiety could also be possible. Experimentally demonstrating that the enzyme is stereoselective was not possible, however, since the vomilenine isomers could not be separated using the established method^24^ and monitored over time in the LC‐based assay (Figure S9). Notably, ajmaline does not equilibrate in a similar fashion to vomilenine, thus suggesting that further modifications of the scaffold, presumably reduction of the C19=C20 double bond, increase the energy barrier between the C‐21 epimers.

The minor enzymatic product of VH was identified as perakine, as evidenced by co‐elution with an authentic standard of perakine (Figure 3 A), along with ^1^H and ^13^C NMR analysis (Table S2, Figure S6), although limited availability of vomilenine precluded the determination of kinetic data for this reaction. Perakine has long been known to be a possible product of vomilenine, since the introduction of a hydroxy group at C‐21 allows opening of the ring via the newly formed hemiaminal. The resulting amine can then undergo a Michael addition to form perakine (Scheme 1). However, while this isomerization from vomilenine to perakine can occur non‐enzymatically, harsh chemical conditions and extended reaction times are required.^25^ It has also been shown that formation of the perakine‐derived product raucaffrinoline is observed only when vomilenine is incubated with crude *Rauwolfia* enzyme mixtures,^26^ thus suggesting that the conversion of vomilenine into perakine is enzymatically catalyzed. However, the enzyme responsible for this conversion has never been identified.

**Scheme 1:**
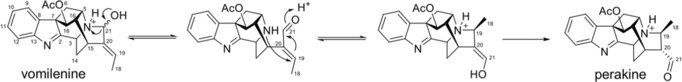
Proposed reaction mechanism for the isomerization of vomilenine to perakine.

A series of control experiments were performed to determine whether VH in fact catalyzes this redox‐neutral isomerization. Isolated vomilenine was not converted into perakine in the assay buffer without the presence of VH (buffer only) or with microsomes lacking VH (Figure 3 B), even over a wide range of pH values from 2.5 to 12.5 (Figure S4B). Formation of perakine was measured in a series of buffers at fixed ionic strength under saturating vinorine concentrations, thus demonstrating that VH is active over a large range of pH values (6.5 to 8.5; Figure S4). NADPH is not required for the reaction, which consistent with the fact that this is a non‐oxidative reaction (Figure S10). The optimal pH for the isomerization of vomilenine to perakine is approximately 4.5, which is unusual for a CYP (Figure S4). VH does not accept perakine as a substrate, thus suggesting that the formation of perakine from vomilenine is directional. The relative yield of perakine is higher when vinorine is used as a substrate (Figure 3 A), compared to when vomilenine is used as a substrate (Figure 3 B). This could suggest that keeping vomilenine in the active site of the CYP is important for more efficient rearrangement of the hydroxylated intermediate. Collectively, our data suggest that in addition to hydroxylating vinorine to form vomilenine, VH also catalyzes the non‐oxidative isomerization of vomilenine to perakine. While it is not clear how VH catalyzes this reaction, we hypothesize that acidic or basic residues within the substrate recognition site of the CYP facilitate either the ring opening or the subsequent Michael addition to form perakine. The reaction could also proceed via an azetidine intermediate; however, modeling studies suggest that this molecule may be too sterically congested, therefore we favor the stepwise mechanism shown in Scheme 1. Since assays with perakine indicated that the isomerization reaction is irreversible, and NMR analysis of the perakine product indicated that no enol is present, we predict that tautomerization of the enol to the aldehyde is likely to be irreversible (solid arrow, Scheme 1). The equilibria of the other steps shown in Scheme 1 are not known. This enzymatic isomerization showed a strong pH dependence, with a pH optimum at pH 4.5 and low efficiency above pH 7 (Figure S4). The p*K*_A_ value of ajmaline is 8.2,^27^ and the structurally similar vomilenine likely has a similar p*K*_A_ value. We hypothesize that enzymatic protonation of the nitrogen may play an important role in facilitating opening of the aminal moiety as the first reaction step (Scheme 1), although the lack of structural data for plant CYP enzymes makes it challenging to design mutations to test this. A CYP that catalyzes a non‐oxidative reaction is highly unusual: to the best of our knowledge, only a single plant CYP that catalyzes a dehydration reaction^28^ along with a fungal CYP that catalyzes an unexpected terpene cyclase reaction have been reported.^29^ It is likely that the inherent reactivity of the vomilenine substrate, along with the availability of an enzyme with a binding pocket that could (even weakly) bind vomilenine, evolved to perform the relatively simple acid–base catalysis to form the perakine product, thereby generating an additional branch of alkaloids.

The dual catalytic function of VH suggests that this enzyme may have a double function in MIA biosynthesis in *Rauwolfia*. While the rates indicated by the in vitro assays suggest that it most efficiently delivers vomilenine as the intermediate in the ajmaline pathway, the enzyme also gives rise to perakine, which is a less abundant alkaloid in *Rauwolfia* spp. The expression profile of VH is consistent with a role in the production of both ajmaline and perakine. VH, like other ajmaline biosynthetic enzymes, is highly expressed in root tissues (Figure S7A), but is also found in leaf, where perakine and perakine‐derived alkaloids accumulate (Figure S7B).^30^ Perakine reductase, being expressed abundantly in leaf, captures the perakine product and drives the pathway toward formation of perakine‐derived products in leaves. Vomilenine reductase, which is highly expressed in young root, provides the driving force for the formation of dihydro‐vomilenine and ajmaline in root. Therefore, control of ajmaline and perakine levels may depend on both the dual catalytic function of VH, and on the tissue expression profile of VH and downstream pathway enzymes. The catalytic activity of VH, along with the localization of ajmaline and perakine enzymes, generates a complex metabolic network that allows the production of chemical diversity in a spatially localized manner.

Herein, we report the discovery of vinorine hydroxylase (VH), a cytochrome P450 that hydroxylates vinorine to form vomilenine, a missing step in the biosynthesis of the antiarrhythmic agent ajmaline. Surprisingly, VH also catalyzes the formation of perakine through a non‐oxidative isomerization reaction of vomilenine. This cytochrome P450 thereby extends control of the bifurcation into two pathway branches via an unusual dual catalytic function. This discovery highlights the catalytic versatility of plant CYP enzymes.

## Conflict of interest

The authors declare no conflict of interest.

## Supporting information

As a service to our authors and readers, this journal provides supporting information supplied by the authors. Such materials are peer reviewed and may be re‐organized for online delivery, but are not copy‐edited or typeset. Technical support issues arising from supporting information (other than missing files) should be addressed to the authors.

Supplementary
