# Supplementary material for: Dual Catalytic Activity of a Cytochrome P450 Controls Bifurcation at a Metabolic Branch Point of Alkaloid Biosynthesis in Rauwolfia serpentina
Source: Angew Chem Int Ed Engl. 2017 Jul 12;56(32):9440–4. doi: 10.1002/anie.201705010 (PMC5582599; doi:10.1002/anie.201705010)
Supplement: Supplementary file 1 — Supplementary [file ANIE-56-9440-s001.pdf]

Angewandte Chemie
Eine Zeitschrift der Gesellschaft Deutscher Chemiker

Supporting Information

Dual Catalytic Activity of a Cytochrome P450 Controls Bifurcation at a Metabolic Branch Point of Alkaloid Biosynthesis in *Rauwolfia serpentina*

*Thu-Thuy T. Dang, Jakob Franke, Evangelos Tatsis, and Sarah E. O'Connor**

anie_201705010_sm_miscellaneous_information.pdf

Author Contributions

T.D. Conceptualization: Equal; Formal analysis: Lead; Investigation: Lead; Methodology: Lead; Writing—original draft: Lead
J.F. Conceptualization: Supporting; Investigation: Supporting; Methodology: Lead; Writing—review & editing: Equal
E.T. Formal analysis: Supporting; Investigation: Supporting; Methodology: Supporting. S.E.O. Conceptualization: Equal; Formal analysis: Supporting; Methodology: Supporting; Writing: Equal.

Supporting information

Table of Contents

SI-1 – Methods **2**

SI-1.1 Plants and chemicals 2

SI-1.2 Generation of self-organizing maps 2

SI-1.3 Phylogenetic analysis 2

SI-1.4 Expression vector construction, protein production, and purification 2

SI-1.5 Yeast culture, preparation of microsomes and immunoblot analysis 2

SI-1.6 Enzyme assays 3

SI-1.7 Gene expression and alkaloid accumulation in different plant organs 3

SI-1.8 LC-MS/MS analysis 3

SI-1.9 NMR analysis 3

SI-2 Supporting figures **4**

Figure S1 Self-organizing map of *R. serpentina* transcriptomic data 4

Figure S2 Substrates tested with 5437 for substrate specificity 5

Figure S3. Western blot showing the expression of CPR (A) and 5437 (B) in *Saccharomyces cerevisiae* harbouring *pESC-Leu2d::CPR* (CPR) and *pESC-Leu2d::5437/CPR* (CPR/5437) 6

Figure S4. pH profiles of VH enzymatic activity under saturating (Vmax) substrate conditions 7

Figure S5. Steady-state enzyme kinetics of RsVH in total microsomal protein extracts of *S. cerevisiae* 8

Figure S6. NMR spectra of RsVH products 9

Figure S7. Relative alkaloid accumulation (A) and transcripts levels of genes involved in MIA biosynthetic pathway in *R. serpentina* organs (B) 18

Figure S8. Key NOE correlations for determination of C-21 stereochemistry of vomilenine in different solvents 19

Figure S9. LC conditions that were used to attempt to separate vomilenine isomers 20

Figure S10. Isomerization of vomilenine to perakine is independent of NADPH 21

SI-3 Supporting tables **22**

Table S1. NMR spectral data for vomilenine in $CDCl_3$, CD_3OD and DMSO-d_6 in comparison with literature data 22

Table S2. NMR spectral data for perakine in $CDCl_3$ in comparison with literature data 24

Table S3. Primers used to construct pESC-Leu2d expression vectors 26

Reference for supporting information 27

SI-1. Methods

SI-1.1 Plants and chemicals. *R. serpentina* seeds were a gift from Dr. Subhash Hiremath, Karnataka University, India. The seeds were germinated at 28/22°C light/dark in a growth chamber with a photoperiod of 16h. The 4-6 weeks plantlets were transferred to individual pots and grown in the greenhouse at the same temperature and light conditions. Vinorine and perakine were purchased from Northernchem Inc. (Niagara Falls, Ontario, Canada). All other chemicals were of analytical grade from Sigma Aldrich.

SI-1.2 Generation of self-organizing maps. Publicly available transcriptomic data of seven different organs of Rauwolfia was filtered for contigs with FPKM (fragments per kilobase of exon per million fragments mapped) expression values higher than zero for more than half of the organs (FPKM expression values of zero for more than half of the treatments or with zero expression variance across the samples were removed). Self-organizing maps were applied and visualized in R (RStudio 1.0.136, RStudio, Inc) with the Kohonen package[1] as reported before.[2] The map was assigned to give about 50 contigs per node. Cytochrome P450 candidates that are in the same nodes or neighbouring nodes that have similar expression patterns with previously reported genes were selected for cloning and testing for activity.

SI-1.3 Phylogenetic analysis. Unrooted neighbour-joining phylogenetic tree for CYP candidates from this study and other reported CYPs from other organisms were performed using the Geneious Tree Builder program in the Geneious software package (Biomatters). Abbreviations and accession numbers: *R. serpentina* CYP5437, KY926696; *C. roseus* cytochrome P450 (CYP71D1V1), AEX07770.1; *C. roseus* CrCYP71BT1, AHK60840.1; *C. roseus* CYP71AY1, CrCYP71Y1, AHK60849.1; *Solanum pennellii* isoflavone 2'-hydroxylase-like, SpFH, XM_015218869.1; *Theobroma cacao* cytochrome CYP71D9, Tc71D9, XP_017975886; *C. roseus* geraniol 10-hydroxylase, CrG10H, Q8VWZ7.1; *Nicotiana tabacum* CYP82M1v4, ABC69375.1; *C. roseus* CYP72A224 7-deoxyloganic acid 7-hydroxylase, Cr7DLH, AGX93062.1; *Papaver somniferum* cytochrome P450 CYP82X1, PsCYP82X1; AFB74614.1; *P. somniferum* cytochrome P450 CYP82Y1, PsCYP82Y1, AFB74617.1; *P. somniferum* cytochrome P450 CYP82X2, PsCYP82X2, AFB74617.1, AFB74616.1; *C. roseus* tabersonine 16-hydroxylase, CrCYP71D12, FJ647194.1; *Salvia miltiorrhiza* cytochrome P450 CYP82V2, SmCYP82V2, AJD25203.1; *C. roseus* secologanin synthase, CrSLS, Q05047.

SI-1.4 Expression vector construction, protein production, and purification. The full-length coding region of CYPs candidates were amplified using cDNA derived from total root RNA of *R. serpentina* using Takara Ex Taq DNA polymerase (Clontech), and primer sets listed in Supplemental Table 3. For heterologous expression of Flag-tagged CYPs in yeast (*Saccharomyces cerevisiae*), the full-length coding regions of CYPs were cloned into SpeI restriction sites of the dual plasmid *pESC-Leu2d::CPR*[3] yielding *pESC-Leu2d::CYP/CPR* using the In-fusion cloning system (Takara Clontech). The protease-deficient yeast strain YPL 154C:Pep4KO was transformed with *pESC-Leu2d::CYP/CPR*. Yeast harboring *pESC-Leu2d::CPR* was used as the negative control.

SI-1.5 Yeast culture, preparation of microsomes and immunoblot analysis. For routine yeast culture, the transgenic yeast strain was inoculated in 2 mL of synthetic complete (SC) medium lacking leucine (SC-Leu) containing 2% (w/v) glucose. The inoculum was cultured overnight at 30°C and 250 rpm. The culture was subsequently diluted 100-fold to an OD of 0.05 in SC-Leu supplemented with 2% (w/v) glucose and cultured for 16 h. Yeast was then harvested and sub-cultured for 24 h in SC-Leu containing 2% (w/v) galactose to induce recombinant protein production. Yeast cells were harvested by centrifugation and lysed for 5 min using a micro-beadbeater and 500-μm diameter glass beads in TES-B (0.6 M sorbitol in

TE) buffer at half of the maximum instrument speed and subsequently centrifuged at 11,000 g for 10 min at 4°C. The supernatant was then transferred to a new tube and centrifuged at 125,000 g for 90 min at 4°C. Finally, the pellet containing microsomes was resuspended with TEG buffer (20% (v/v) glycerol in TE). Recombinant enzymes were detected by immunoblot analysis using α-FLAG M2 and α-c-Myc antibodies (Genscript) detected with SuperSignal West Pico Chemiluminescent Substrate (ThermoFisher Scientific). Co-expression of 5437 and CPR was confirmed by immunoblot analysis of microsomal fractions prepared from *S. cerevisiae* cultures harboring the *pESC-Leu2d::CPR/5437* vector using FLAG and c-Myc antibodies to detect epitope-tagged recombinant proteins (Supplemental Figure S3).

SI-1.6 Enzyme assays. Standard enzyme assays were performed at 30°C for 30 min in 100 µL of 100 mM HEPES-NaOH, pH 6.5, containing 0.5 mg of total microsomal proteins, 10 µM substrate and 100 µM NADPH on a gyratory shaker with gentle agitation (60 rpm). Reactions were stopped by adding 800 µL methanol. A variety of alkaloids was used to test substrate specificity. The reaction mixture was extracted twice with methanol to precipitate and remove proteins. The supernatant was reduced to dryness and redissolved with appropriate solvent for LC-MS/MS analysis. Steady-state enzyme kinetics was determined by varying the concentration of vinorine between 0 and 100 µM at a fixed concentration of 100 µM NADPH. Kinetic constants were calculated by fitting initial velocity versus substrate concentration to the Michaelis-Menten equation using GraphPad Prism 5.0 (GraphPad Software). A set of constant composition buffers with a constant ionic strength (I = 0.20) of acetic acid (pKa'= 4.64), Tris (pKa'=3.62) and Bis-Tris (pKa' = 8) following the procedure reported before[4] covering the pH from 4.5 to 9.5 was used to study effects of pH on hydroxylation and isomerization activity of VH. Na_2HPO_4 buffer was used to cover pH from 10.5 to 12. Isomerization of vomilenine to perakine were performed at 30°C overnight (16 hrs) in 100mM mixed buffer system pH 4.5 with 0.5 mg of total microsomal proteins and 10 µM vomilenine on a gyratory shaker with gentle agitation (60 rpm).

SI-1.7 Gene expression and alkaloid accumulation in different plant organs. Different organs of *R. serpentina* were collected and flash frozen in liquid N_2. Alkaloids and total RNA was extracted from each organ as described previously. [5]

SI-1.8 LC-MS/MS analysis. Enzyme assays and alkaloid profiling of different organs were analysed by Ultraperformance liquid chromatography (UPLC) on a Waters Acquity UPLC system (Milford, MA). For kinetic studies, chromatography was performed on a BEH Shield RP18 (50 x 2.1 mm; 1.7 µm) column at a flow rate of 0.6 $mL.min^{-1}$. The column was equilibrated in solvent A (0.1% formic acid) and the following elution conditions were used: 0 min, 5% B (100% acetonitrile), from 0 to 3.5 min, 35% B; from 3.5 min to 3.75 min, 100%B; 3.75 min to 4.75, 100%B; 4.75 to 6 min, 5% B to re-equilibrate the column. Alternatively, the column was equilibrated in solvent A1 (0.1% NH_4OH) and the following elution conditions were used: 0 min, 5%B; from 0 to 17.5 min, 65% B; from 17.5 min to 18 min, 100%B; 18 min to 20, 100%B; 20.1 to 23 min, 0% B to re-equilibrate the column. The basic mobile phase improves the peak shape of perakine.

SI-1.9 NMR analysis. A scaled-up standard VH reaction was performed to produce sufficient product amounts of vomilenine and perakine for NMR analysis. The reaction was terminated by the addition of an equal volume of methanol and precipitated protein was removed by centrifugation. Product purification from the concentrated supernatant was performed by Dionex UltiMate™ 3000 Semi-Preparative System (Thermo Fisher Scientifics) with XBridge 5µm BEH C18 ODB™ 130 Å, 10 x 250 mm at a flowrate of 5 $mL.min^{-1}$. The column was equilibrated in solvent A1 (0.1% NH_4OH) and the following elution conditions were used: 0 min, 20%B; from 0 to 15.1 min, 70% B; from 15.1 min to 17.5 min, 95%B; 17.5 min to 18, 95%B; 18 to 21 min, 20% B to re-equilibrate the column. Approximately 0.2 to 0.5 mg of each product were independently dissolved in 600 µL of $CDCl_3$ or CD_3OD and subjected to ^1H, ^{13}C,

NOESY (mixing time 0.6 s), COSY, HSQC and HMBC NMR analysis on Bruker Avance III 400 NMR spectrometer, operating at 400 MHz. Analysis of vomilenine epimers in DMSO-d_6 was performed on a Bruker Avance DRX500 operating at 500 MHz.

SI-2 Supporting figures

Figure S1. Self-organizing map of *R. serpentina* transcriptomic data. Each node represents approximately 50 unigenes with the most similar expression profile. Neighbouring nodes contain unigenes that are related to each other by the similarity of their expression profile. The average expression profiles of genes in the node are plotted within each node. The reported MIA pathway genes depicted in Figure 1 are highlighted in the nodes with bold circle. Green nodes signify the highest quality nodes.

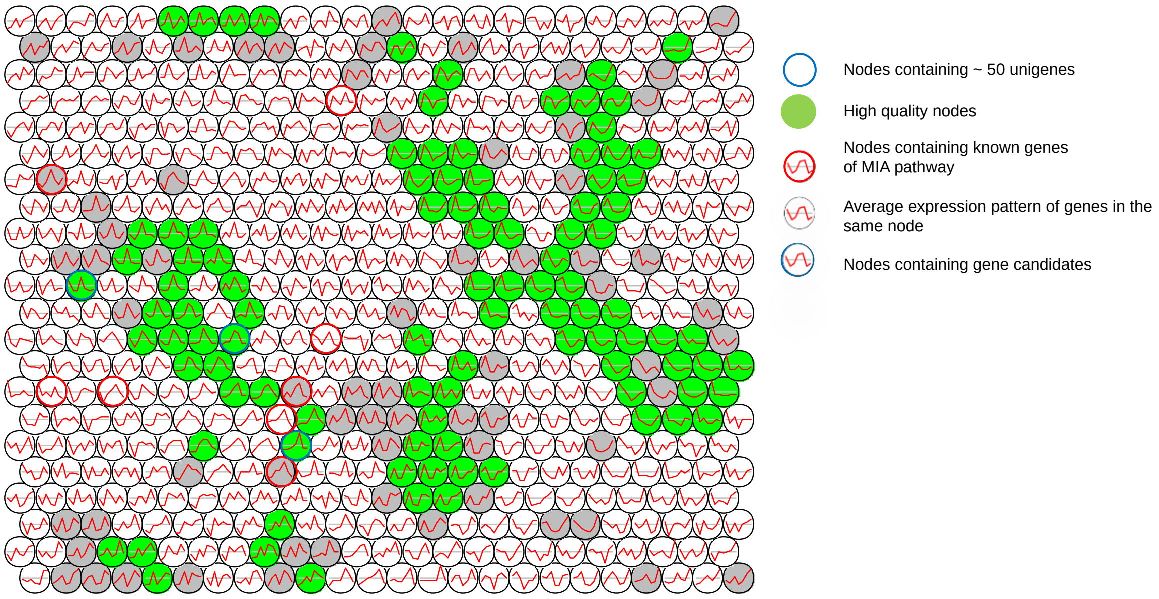

Figure S2. Substrates tested with 5437 for substrate specificity. Substrates are from different subgroups of MIA, including heteroyohimbanes (ajmalicine, tetrahydroalstonine, mayumbine), yohimbanes (corynantheine, yohimbine, rauwolscine), sarpagane (polyneuridine aldehyde), and ajmalanes (vinorine, vomilenine, ajmaline).

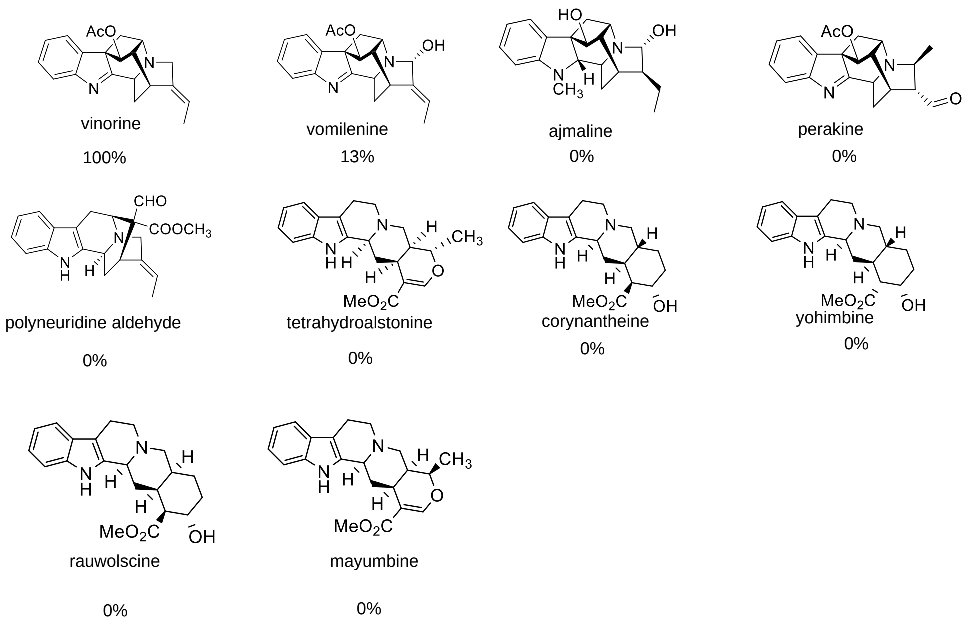

Figure S3. Western blot showing the expression of CPR (A) and 5437 (B) in *Saccharomyces cerevisiae* harbouring *pESC-Leu2d::CPR* (CPR) and *pESC-Leu2d::5437/CPR* (CPR/5437). Protein expression was induced by adding galactose. 5437 and CPR recombinant proteins were detected using α-FLAG (CYP) and α-c-Myc (CPR) antibodies.

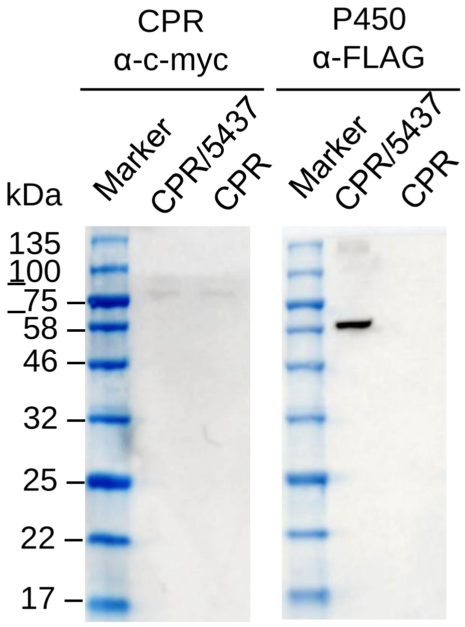

Figure S4. pH profiles of VH enzymatic activity under saturating (Vmax) substrate conditions. A. The conversion of vinorine to vomilenine (black line) and perakine (dotted line) by VH under different pH conditions. B. The conversion of vomilenine to perakine can only be observed when RsVH is present (black line). The incubation of vomilenine with different pH conditions with microsomes without enzyme RsVH (dotted line) did not yield any perakine above the background level.

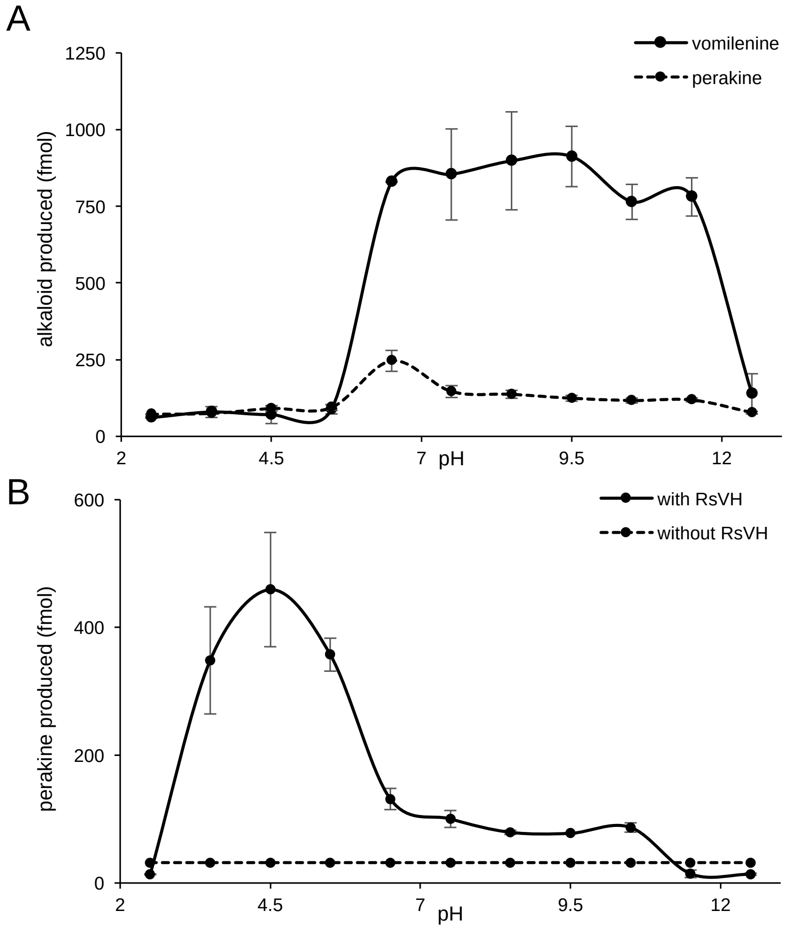

Figure S5. Steady-state enzyme kinetics of RsVH in total microsomal protein extracts of *S. cerevisiae*. Various concentrations of vinorine (A) and NADPH (B) were used as substrates.

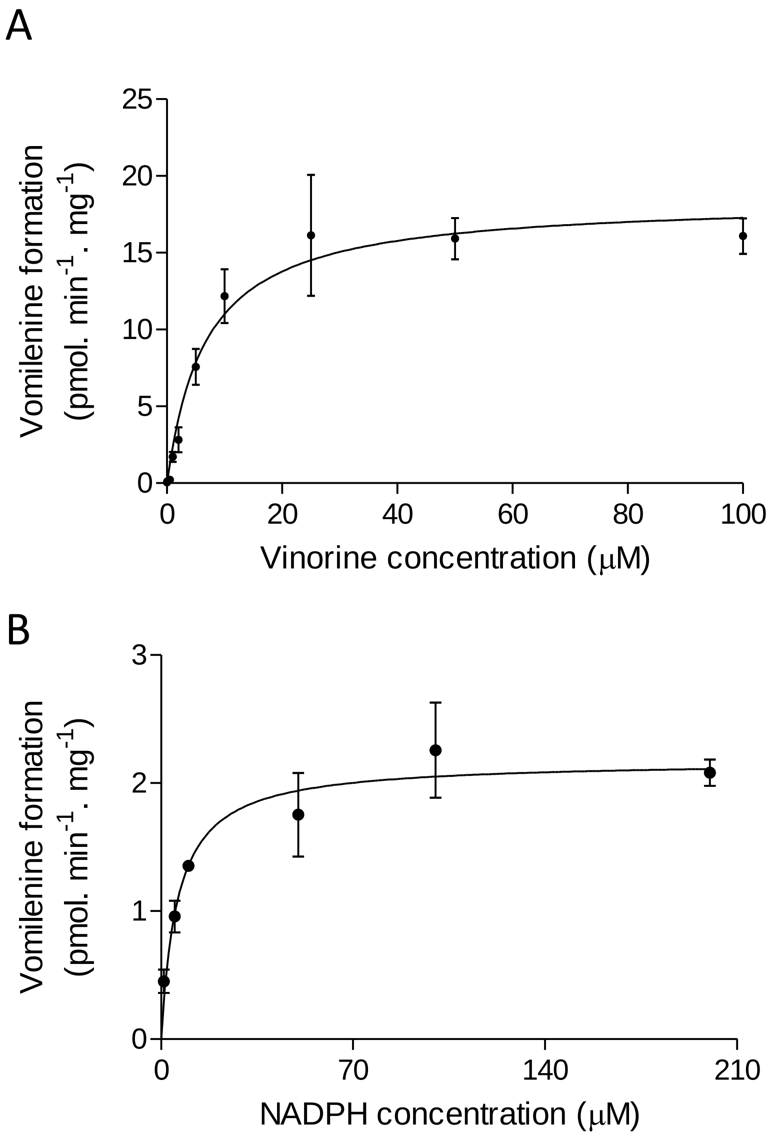

Figure S6. NMR spectra of RsVH products.

^{1}H spectrum of authentic perakine standard ($CDCl_3$, 400 MHz, 298 K).

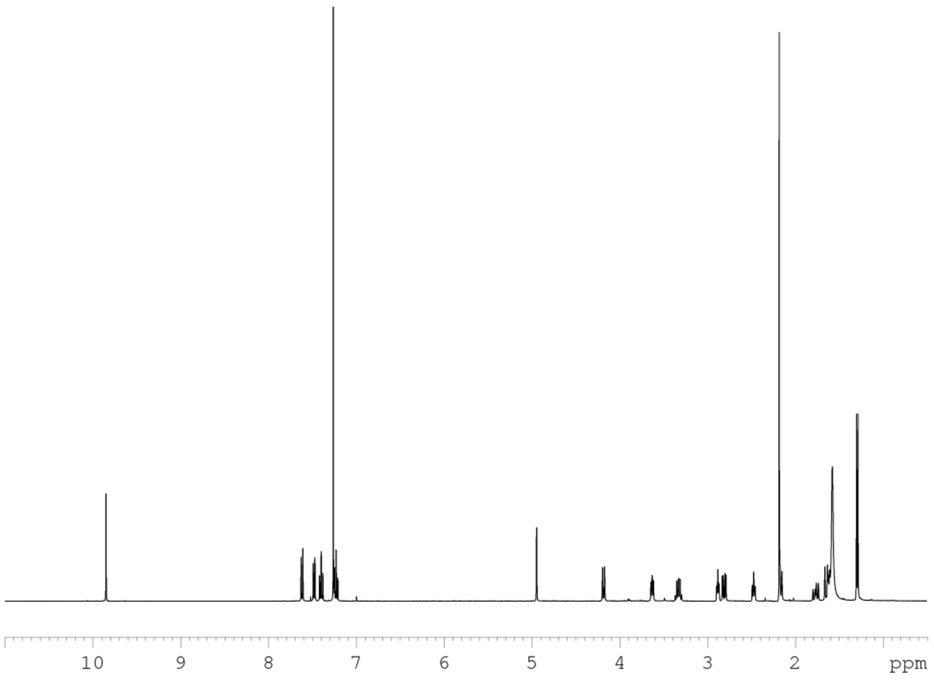

^{13}C spectrum of authentic perakine standard ($CDCl_3$, 100 MHz, 298 K).

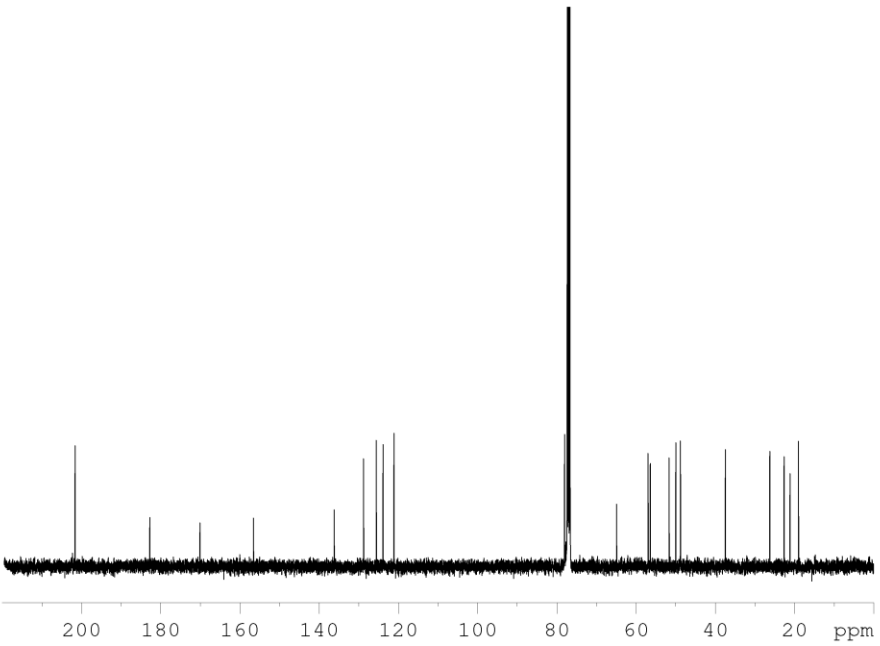

^{1}H spectrum of perakine from enzymatic assay ($CDCl_3$, 400 MHz, 298 K).

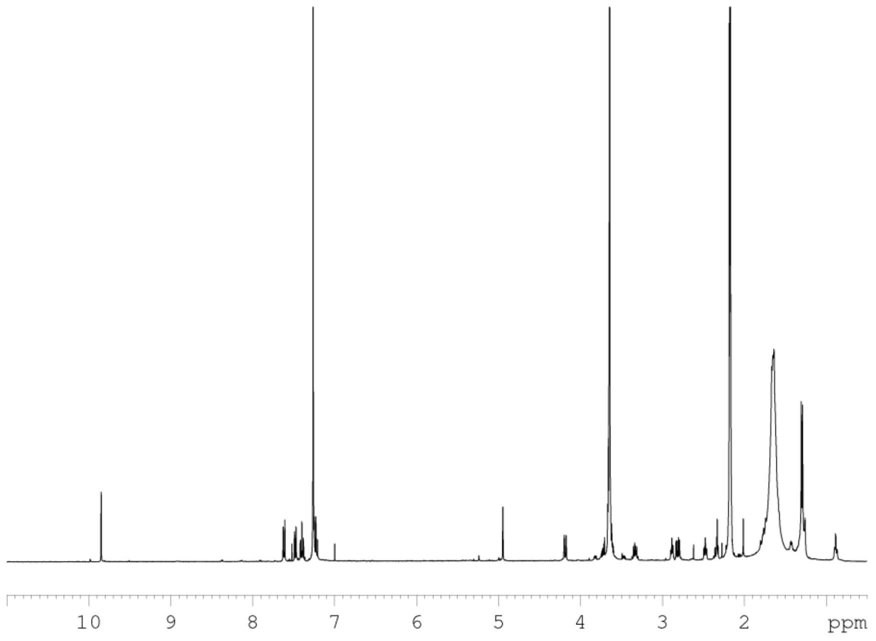

^{13}C spectrum of perakine from enzymatic assay ($CDCl_3$, 100 MHz, 298 K).

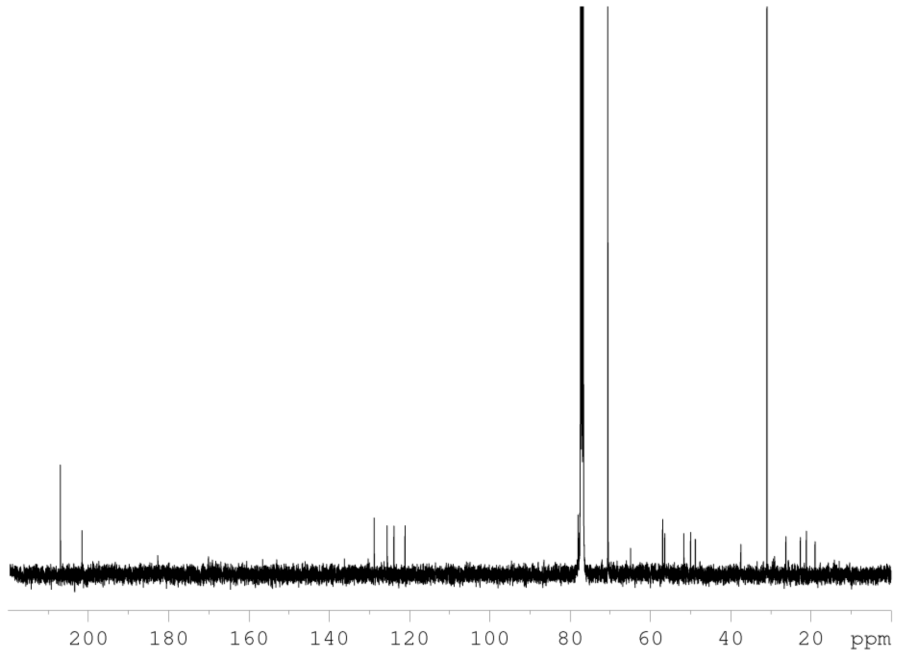

^1H spectrum of vomilenine from enzymatic assay ($CDCl_3$, 400 MHz, 298 K).

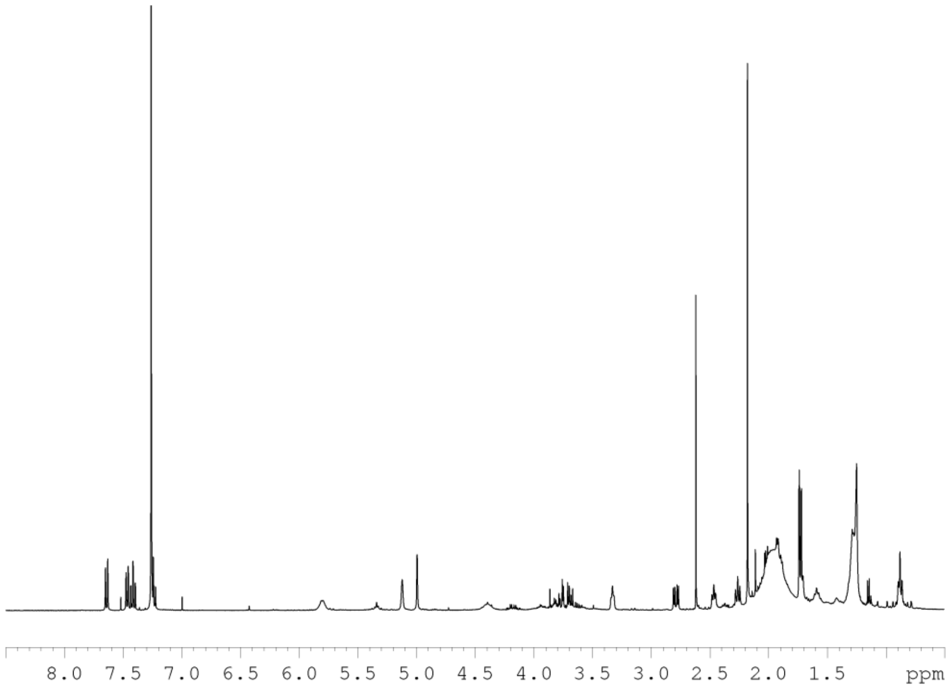

COSY spectrum of vomilenine from enzymatic assay ($CDCl_3$, 400 MHz, 298 K).

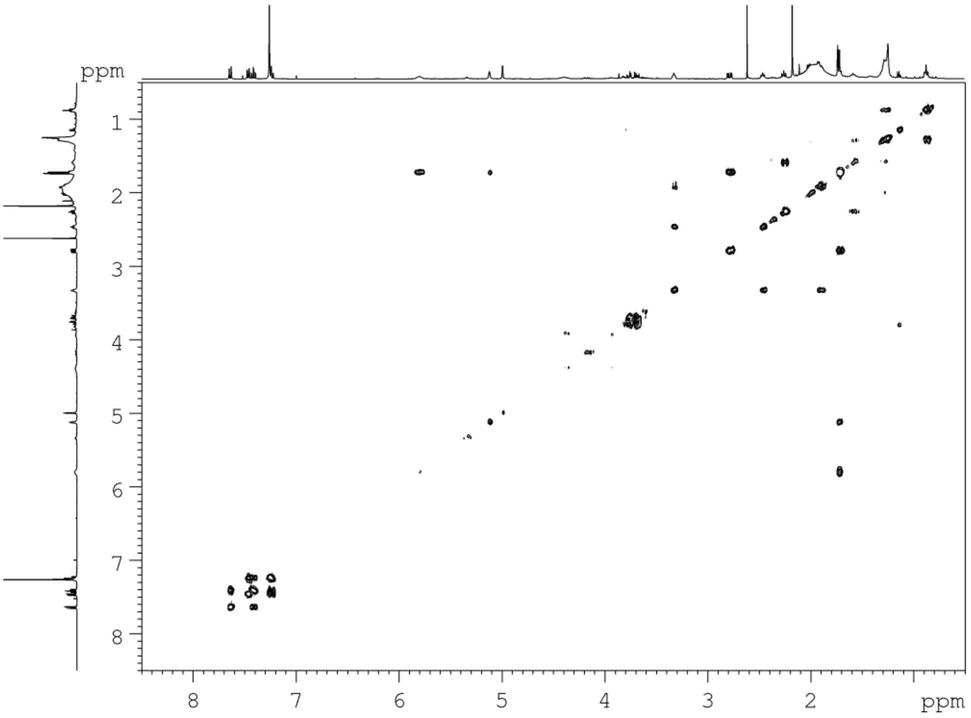

HSQC spectrum of vomilenine from enzymatic assay ($CDCl_3$, 400 MHz, 298 K).

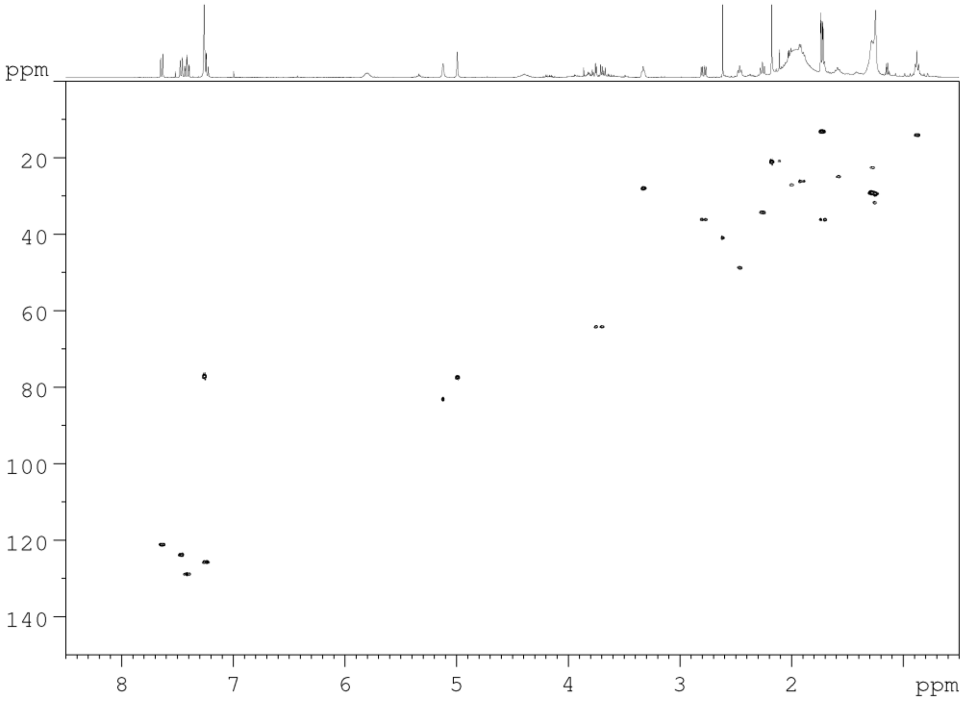

HMBC spectrum of vomilenine from enzymatic assay ($CDCl_3$, 400 MHz, 298 K).

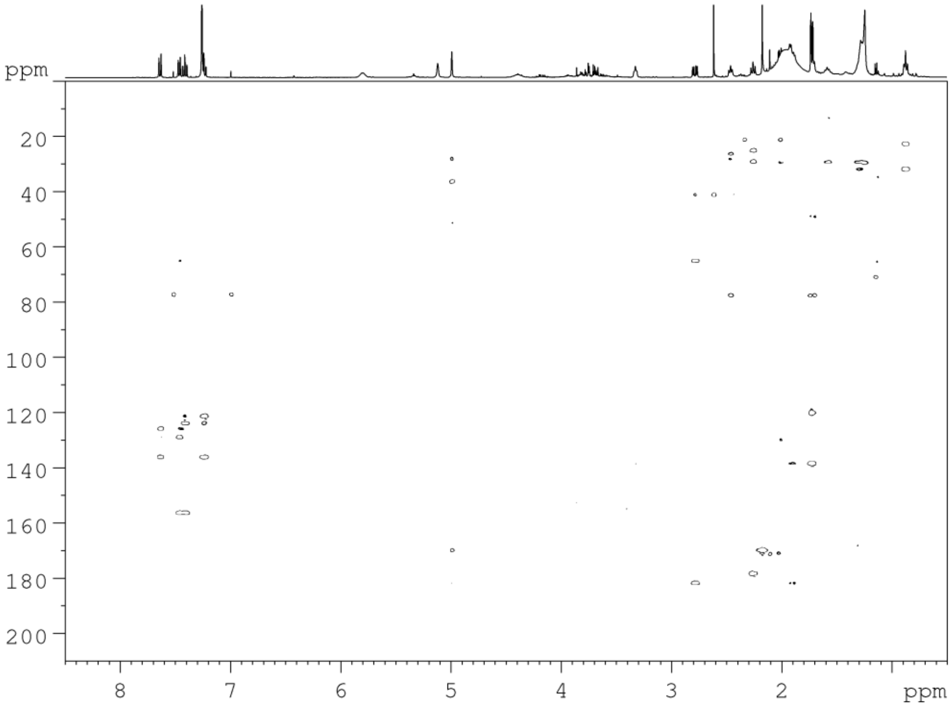

NOESY spectrum of vomilenine from enzymatic assay ($CDCl_3$, 400 MHz, 298 K).

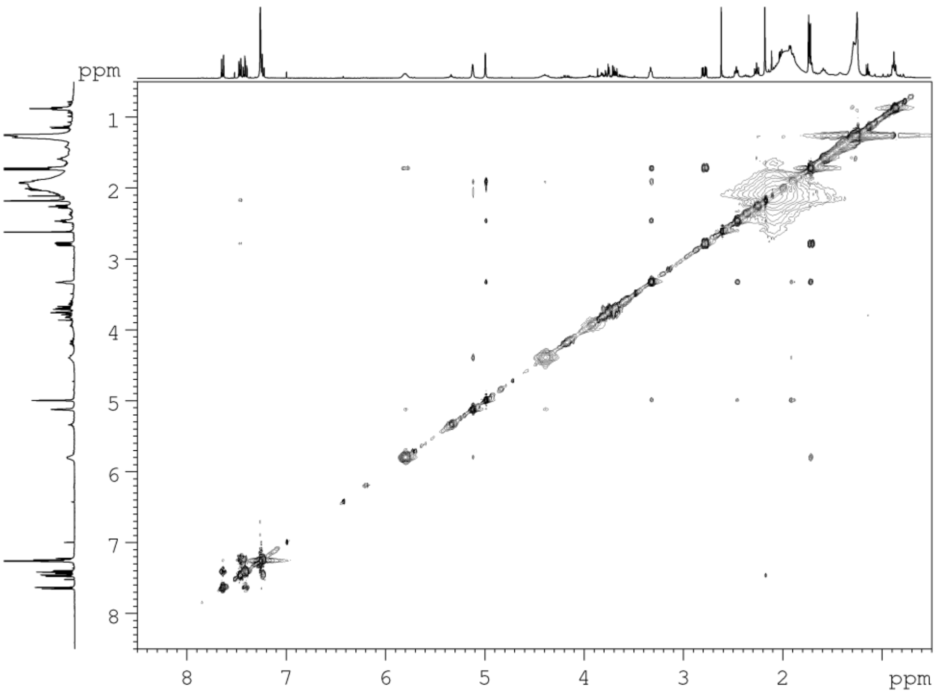

^{1}H spectrum of vomilenine from enzymatic assay (MeOD, 400 MHz, 298 K).

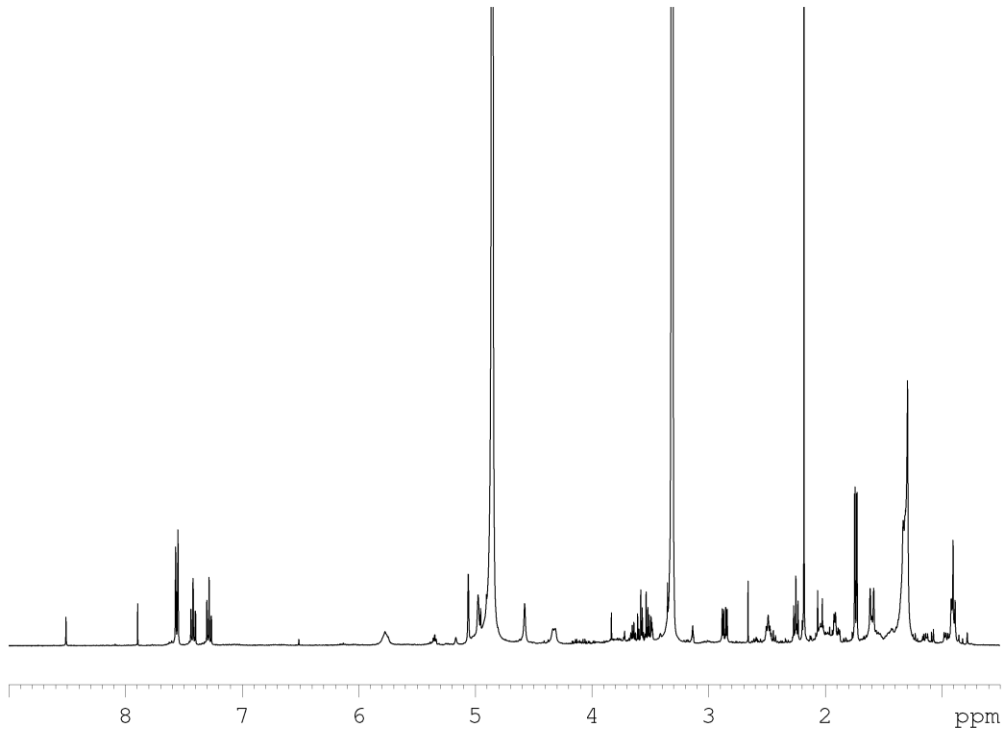

HSQC spectrum of vomilenine from enzymatic assay (MeOD, 400 MHz, 298 K).

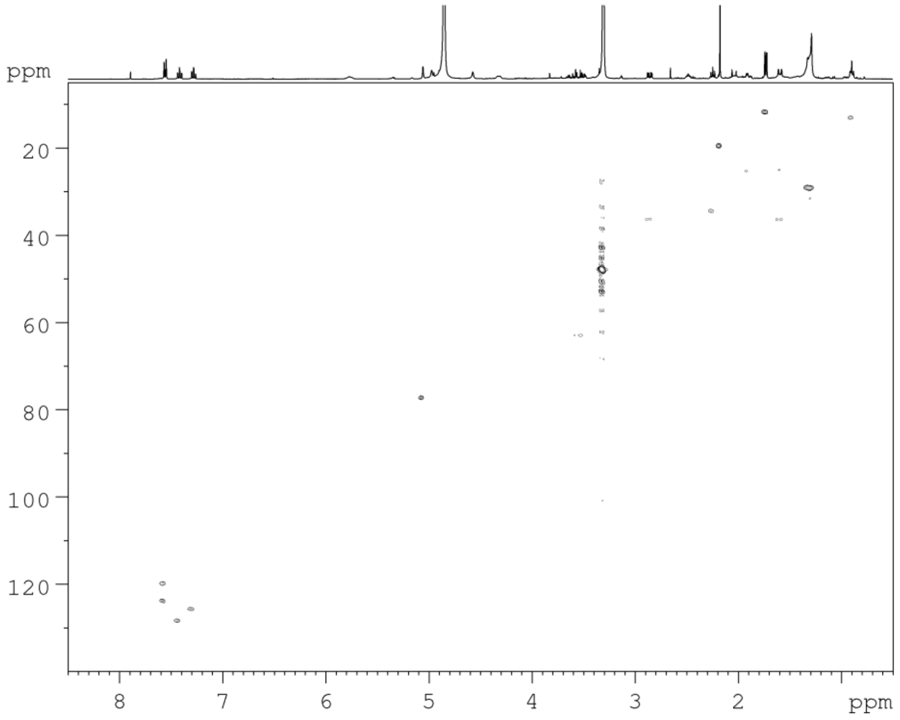

NOESY spectrum of vomilenine from enzymatic assay (MeOD, 400 MHz, 298 K).

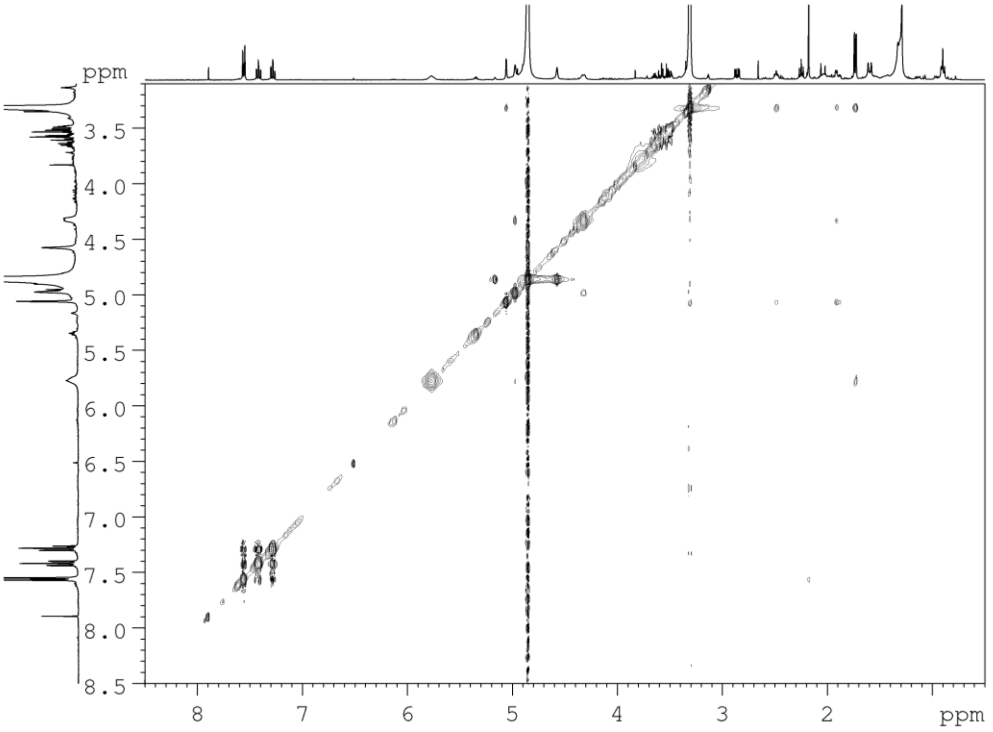

COSY spectrum of vomilenine from enzymatic assay (MeOD, 400 MHz, 298 K).

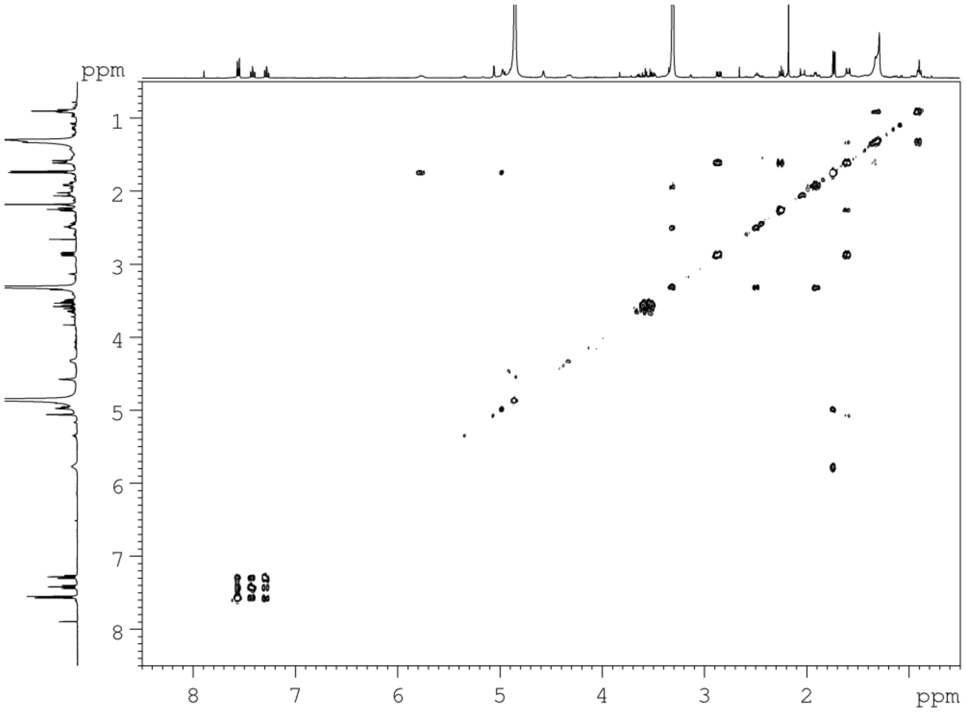

HMBC spectrum of vomilenine from enzymatic assay (MeOD, 400 MHz, 298 K).

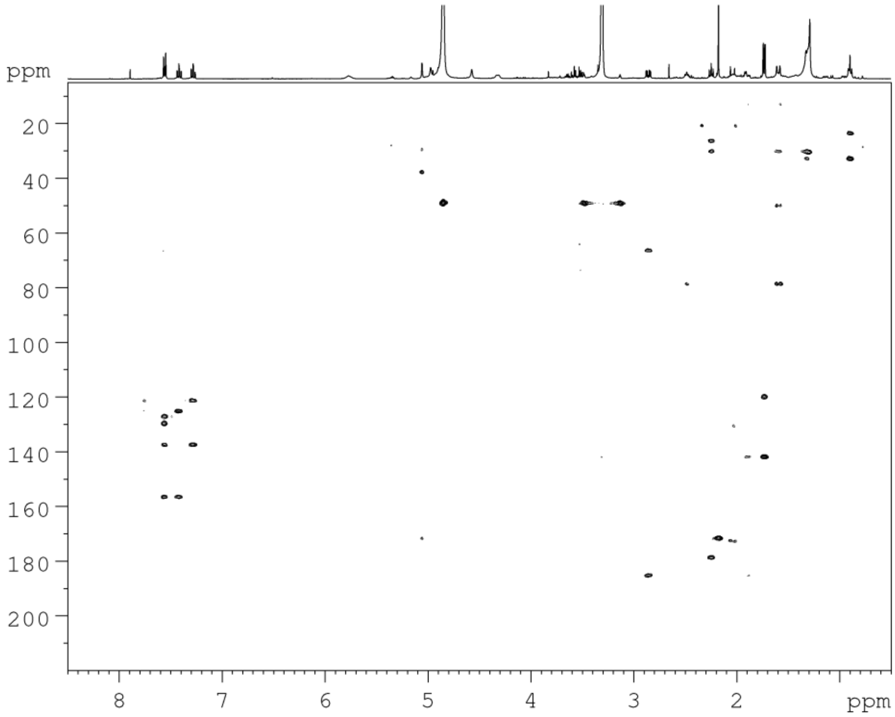

^1H spectrum of vomilenine from enzymatic assay (DMSO-d_6, 500 MHz, 298 K).

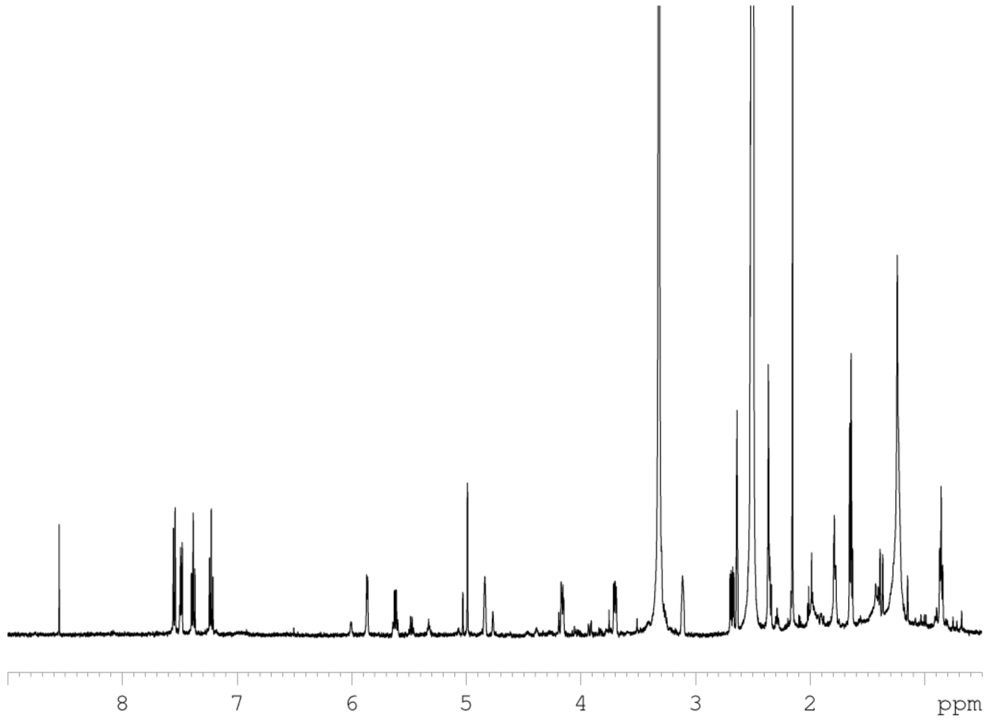

COSY spectrum of vomilenine from enzymatic assay (DMSO-d_6, 500 MHz, 298 K).

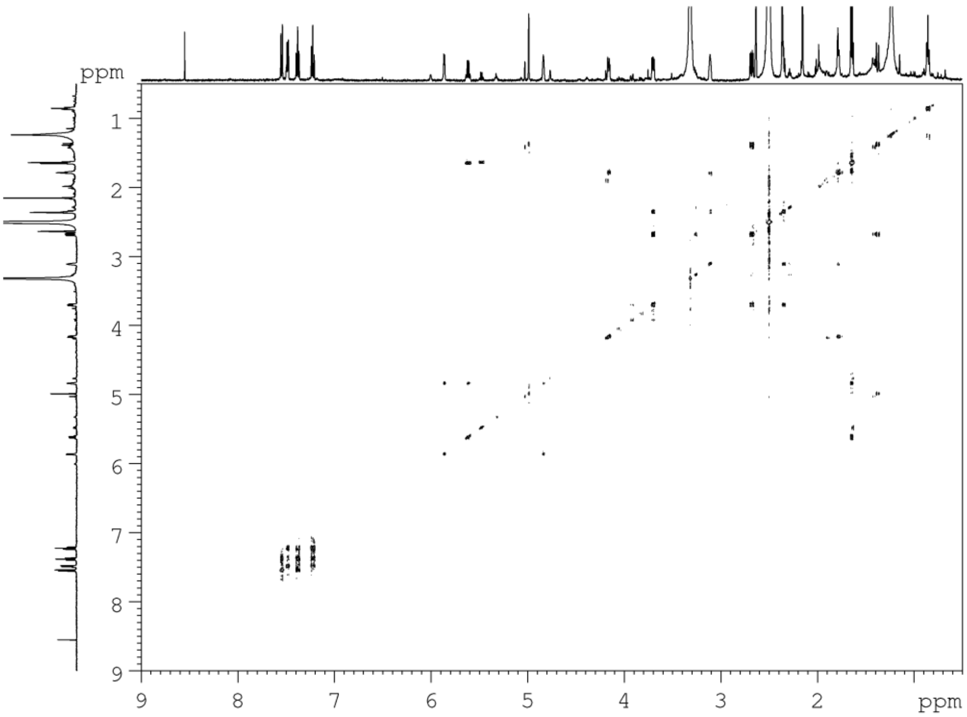

NOESY spectrum of vomilenine from enzymatic assay (DMSO-d$_6$, 500 MHz, 298 K).

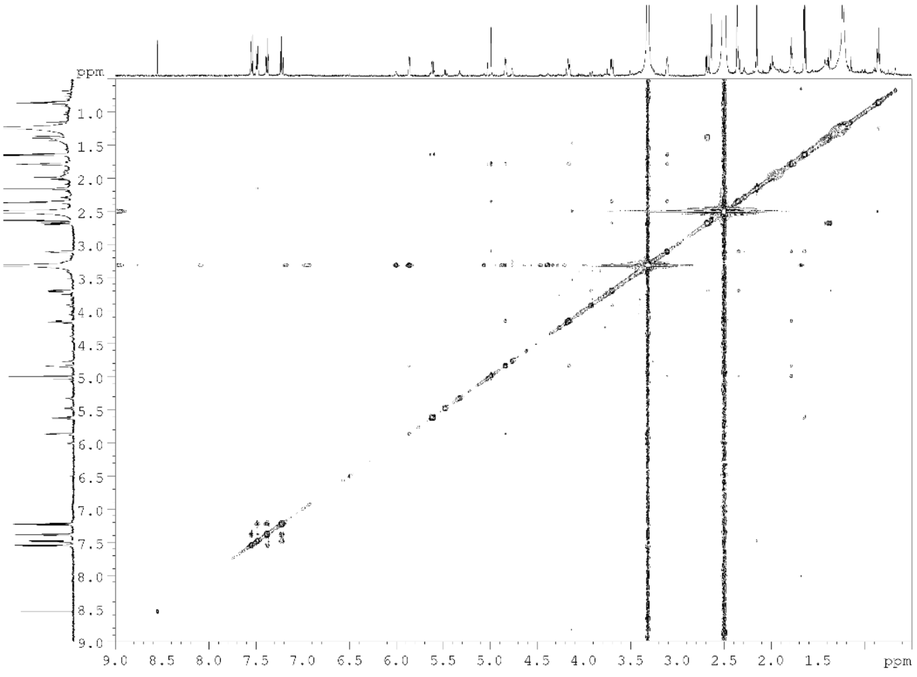

HSQC spectrum of vomilenine from enzymatic assay (DMSO-d$_6$, 500 MHz, 298 K).

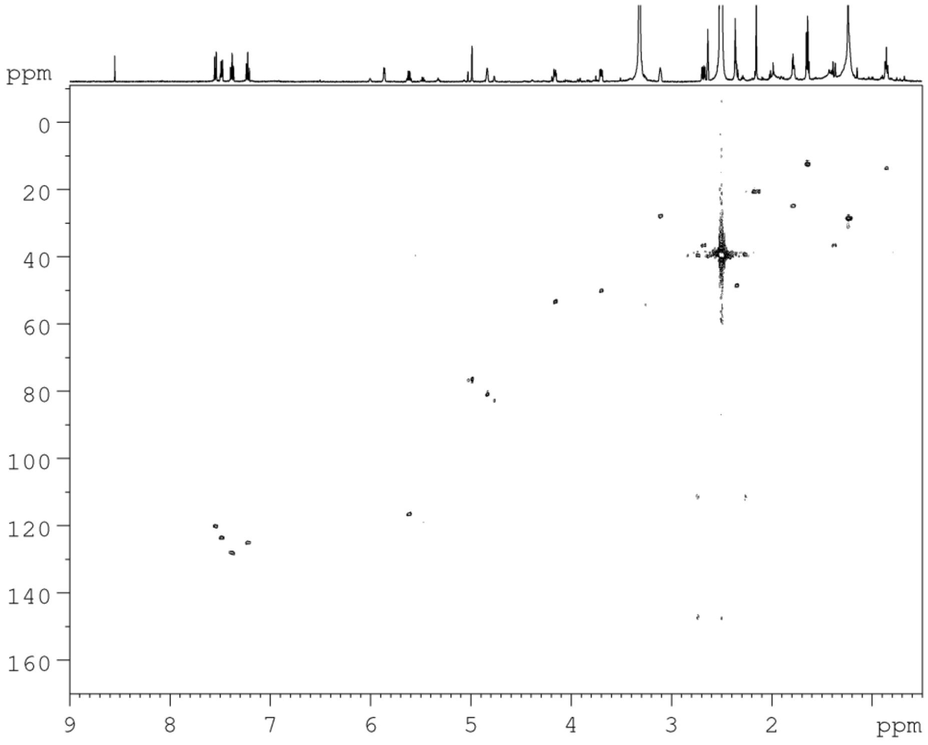

Figure S7. Relative alkaloid accumulation (A) and transcripts levels of genes involved in MIA biosynthetic pathway in *R. serpentina* organs (B). VS, vinorine synthase; VH, vinorine hydroxylase; PR, perakine reductase; VR, vomilenine reductase 1 (see Figure 1).

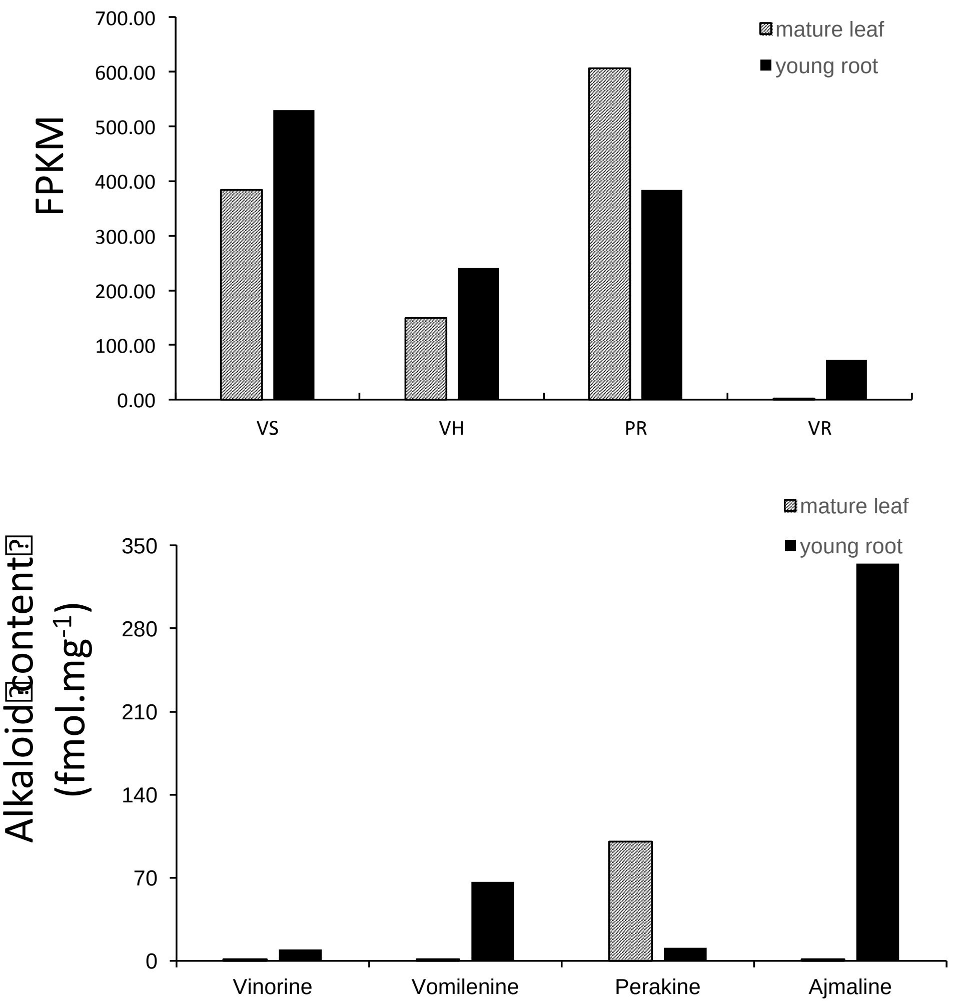

Figure S8. Key NOE correlations for determination of C-21 stereochemistry of vomilenine in different solvents.

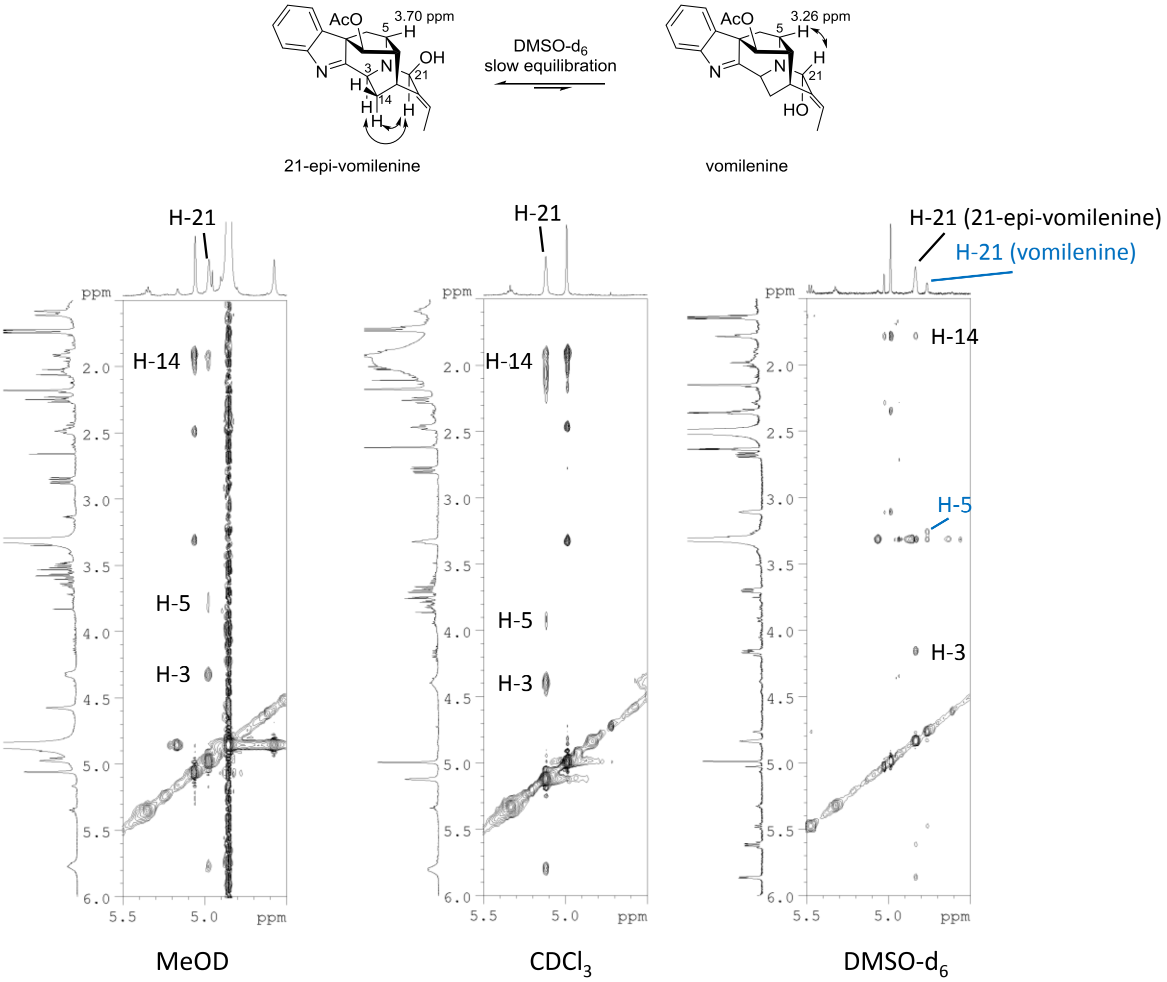

Figure S9. LC conditions that were used to attempt to separate vomilenine isomers. A, 0.1% formic acid) and (B, 0.1% NH_4OH phases) in combination with different concentrations of acetonitrile (ACN). Chromatography was performed on a Lux i-Cellulose 5 (150 x 3.0 mm, 3 µm) column (Phenomenex) with a flow rate of 1 mL/min. Mass spectrometry detection was performed on a Waters Xevo TQ-S mass spectrometer as described in the Methods.

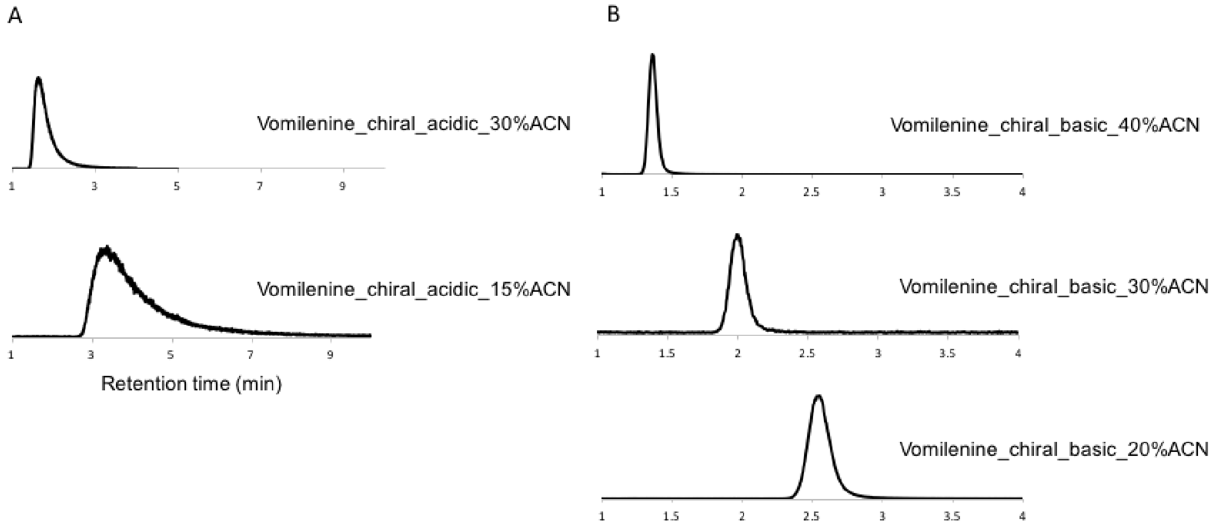

Figure S10. Isomerization of vomilenine to perakine is independent of NADPH. Increasing concentration of NADPH did not result in more production of perakine.

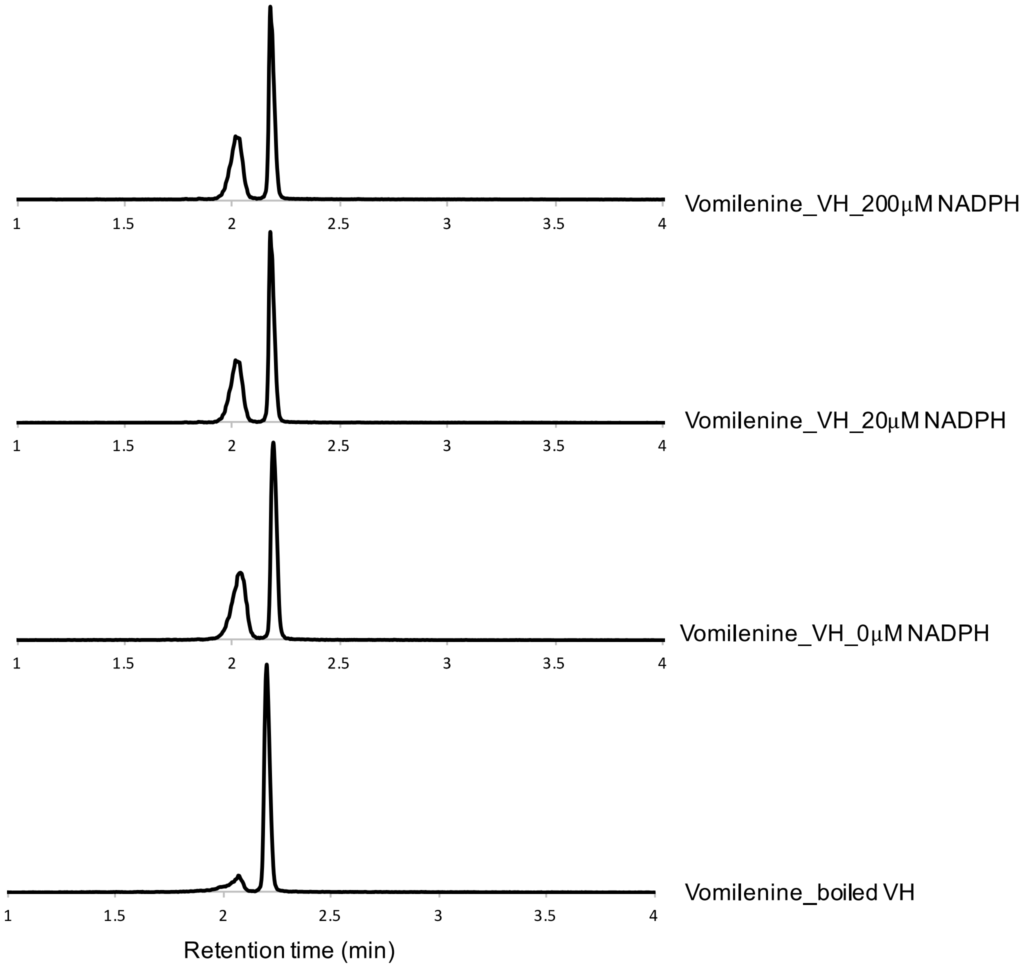

SI3-Supporting tables

Table S1. ^{13}C NMR data of enzymatically produced vomilenine (298 K, 100 MHz)

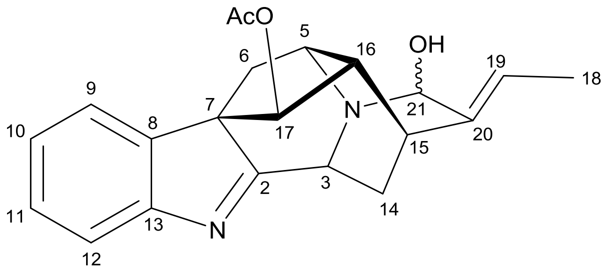

vomilenine

No	This study ($CDCl_3$)	Batista et al. 1995 ($CDCl_3$)	Libot et al. ($CDCl_3$)	This study (MeOD)	This study (21-epi-vomilenine, DMSO-d_6)[a]	This study (vomilenine, DMSO-d_6)[a]
2	181.7	181.5	183.6	185.3	[b]	[b]
3	n.d.	54.1	54.3	n.d.	53.4	53.4
5	n.d.	49.1	50.9	n.d.	50.2	54.3
6	36.2	36.4	36.4	37.7	36.7	36.7
7	65.0	64.5	65.1	66.6	[b]	[b]
8	136.1	136.0	136.1	137.5	[b]	[b]
9	123.8	123.8	123.9	125.0	123.7	123.7
10	125.7	125.8	125.8	127.0	125.1	125.1
11	128.9	128.9	128.9	129.6	128.1	128.1
12	121.2	121.3	121.1	121.1	120.2	120.2
13	156.5	156.5	156.3	156.6	[b]	[b]
14	26.2	26.3	26.3	26.7	25.0	25.0
15	28.0	28.3	28.2	29.6	28.0	28.0
16	48.8	49.0	49.0	50.2	48.5	47.1
17	77.4	79.6	77.5	78.6	76.5	76.8
18	13.2	13.2	13.0	12.8	12.6	12.6
19	120.2	120.0	119.4	119.9	116.7	119.0
20	138.6	128.5	131.0	141.9	[b]	[b]
21	83.1	82.5	82.5	83.4	81.0	82.8
**Me**(CO)O	21.1	21.1	21.1	20.6	20.6	20.6
Me(**CO**)O	169.9	169.9	169.7	171.9	[b]	[b]

[a] Two signal sets are observed in DMSO-d_6, corresponding to 21-epi-vomilenine as the main component and vomilenine as the minor one. Measurements at 500 MHz.
[b] Quaternary carbons not analysed due to insufficient amounts

^{1}H NMR data of enzymatically produced vomilenine (298 K, 400 MHz)

No	**This study ($CDCl_3$)**	**Batista et al. 1995 ($CDCl_3$)**	**This study (MeOD)**	**This study (21-epi-vomilenine, DMSO-d_6)[a]**	**This study (vomilenine, DMSO-d_6) [a]**
3	4.39 (br s) (NOESY)	4.41 (sbr)	4.33 (br d, 7.5) (NOESY)	4.16 (m)	4.18 (m)
5	3.93 (br s) (NOESY) [b]	2.46 (dd, 5.7, 6.1)	3.78 (m) (NOESY)	3.70 (dd, 6.8, 5.3)	3.26 (m)
6	2.79 (dd, 12.2, 4.9)<br>1.71 (m)	2.80 (dd, 12.2, 4.8)<br>1.72 (dd, 6.9, 1.5)	2.86 (dd, 12.0, 5.0)<br>1.59 (d, 12.0)	2.68 (dd, 11.7, 4.9)<br>1.38 (d, 11.8)	2.68 (dd, 11.7, 4.9)<br>1.41 (d, 11.8)
9	7.47 (d, 7.3)	7.47 (d, 7.8)	7.56 (d, 8.0)	7.48 (d, 7.3)	7.49 (d, 7.4)
10	7.24 (ddd, 7.5, 7.5, 1.0)	7.25 (ddd, 7.5, 7.3, 1.0)	7.28 (ddd, 7.6, 7.6, 1.0)	7.22 (ddd, 7.5, 7.5, 1.0)	7.22 (ddd, 7.5, 7.5, 1.0)
11	7.41 (ddd, 7.7, 7.7, 1.2)	7.42 (ddd, 7.7, 7.6, 1.3)	7.42 (ddd, 7.5, 7.5, 1.3)	7.38 (ddd, 7.7, 7.7, 1.2)	7.38 (ddd, 7.7, 7.7, 1.2)
12	7.64 (d, 7.8)	7.64 (d, 7.6)	7.56 (d, 8.0)	7.55 (d, 7.7)	7.55 (d, 7.7)
14	1.90 (m)	1.95 (d)	1.90 (dd, 14.1, 5.0)<br>1.60 (m)	1.79 (m)	1.90 (m)
15	3.33 (t, 5.3) [b]	Not reported	3.32 (m)	3.11 (m)	3.11 (m)
16	2.48 (t, 6.3) [b]	3.33 (sbr)	2.49 (t, 6.3)	2.35 (t, 6.3)	2.29 (t, 6.3)
17	4.99 (s)	4.99 (d)	5.06 (s)	4.99 (d, 0.7)	5.03 (d, 0.7)
18	1.73 (dd, 7.1, 1.7) [b]	1.25 (s)	1.74 (dd, 7.0, 1.7)	1.65 (dd, 7.1, 2.0)	1.63 (m)
19	5.80 (br q, 6.5) [b]	3.90 (sbr)	5.78 (br s)	5.62 (qd, 7.0, 1.8)	5.48 (q, 7.0)
21	5.12 (s)	5.13 (sbr)	4.98 (s)	4.84 (br s)	4.77 (br s)
**Me**(CO)O	2.18 (s)	2.18 (s)	2.18 (s)	2.15 (s)	2.15 (s)
C21-OH	-	-	-	5.86 (d, 4.3)	6.00 (br s)
Indole-NH	-	-	-	8.55 (s)	8.55 (s)

[a] Two signal sets are observed in DMSO-d_6, corresponding to 21-epi-vomilenine as the main component and vomilenine as the minor one. Measurements at 500 MHz.

[b] Despite the differences to the data reported for vomilenine, the chemical shifts are in good agreement with literature reports of similar compounds [6–8]

Table S2. NMR spectral data for perakine in $CDCl_3$ in comparison with literature data.

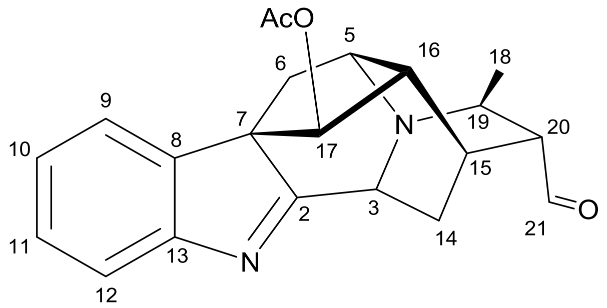

perakine

^{13}C NMR data of enzymatically produced perakine in $CDCl_3$ (298 K, 100 MHz)

C	This study	Batista et al. 1995
2	182.7	182.6
3	56.9	56.9
5	51.6	51.6
6	37.4	37.3
7	64.9	64.8
8	136.2	136.1
9	123.9	123.9
10	125.6	125.6
11	128.8	128.8
12	121.1	121.1
13	156.5	156.5
14	22.6	22.6
15	26.2	26.2
16	48.8	48.7
17	78.0	78.0
18	18.9	18.9
19	49.9	49.9
20	56.4	56.3
21	201.6	201.5
**Me**(CO)O	21.1	21.1
Me(**CO**)O	170.1	170.1

^{1}H NMR data of enzymatically produced perakine in $CDCl_3$ (298 K, 400 MHz)

**H**	**This study**	**Batista et al. 1995**
3	4.18 (d, 9.3)	4.20 (d, 9.1)
5	3.63 (m)	3.64 (dd, 6.7, 5.3)
6	2.81 (dd, 12.0, 5.1)	2.82 (dd, 12.1, 5.3)
	n.d.[a]	1.66 (d, 12.1)
9	7.48 (dd, 7.6, 1.2)	7.49 (dd, 7.2, 1.5)
10	7.23 (ddd, 7.6, 7.6, 1.3)	7.23 (ddd, 7.7, 7.5, 1.1)
11	7.40 (ddd, 7.6, 7.8, 1.2)	7.40 (ddd, 7.8, 7.5, 1.0)
12	7.62 (dd, 7.8, 1.3)	7.62 (d, 7.8)
14	1.78 (m)	1.77 (dddd, 14.8, 9.2, 2.0, 1.0)
	n.d.[a]	1.60 (dddd, 14.8, 5.2, 2.0, 1.0)
15	2.88 (dd, 5.1, 5.1)	2.91 (dd, 5.5, 5.2)
16	2.47 (ddd, 7.4, 5.2, 1.1)	2.48 (ddd, 7.0, 5.5, 1.7)
17	4.94 (d, 1.3)	4.95 (d, 1.7)
18	1.29 (d, 6.7)	1.30 (d, 6.7)
19	3.33 (dq, 9.4, 6.7)	3.34 (dq, 9.3, 6.7)
20	n.d.[b]	2.17 (d, 9.3)
21	9.85 (d, 0.8)	not reported
**Me**(CO)O	2.18 (s)	2.17 (s)

[a] overlapped by residual water signal

[b] overlapped by acetone contamination signal

Table S3. Primers used for the assembly of expression vectors

Primers used to assemble CYP candidates in pESC-Leu2d expression vector

Vector name	Forward primer (5'-3')	Reverse primer (5'-3')	Insert size (bp)
pESC-Leu2d-5437	ACC CTC ACT AAA GGG CGG CCG CAA CCA TGG ACT TAT TAC AAA TCT TA TT	GTC ATC CTT GTA ATC CAT CGA TAC AGG TTC ATA CAA GCA CTT GG CA	1575
pESC-Leu2d-379	ACC CTC ACT AAA GGG CGG CCG CAA CCA TGA TGG AGC TCA TCG TTC TCC TTT TTG	GTC ATC CTT GTA ATC CAT CGA TAC TTC CCC TTT GTA ATC AAC ATG AG	1527
pESC-Leu2d-3375	ACC CTC ACT AAA GGG CGG CCG CAA CCA TGA TGG AGT TCT CTT TCT CCT TTC C	GTC ATC CTT GTA ATC CAT CGA TAC TTT TGC AAG ATG AGG AAC CAG TTT	1503
pESC-Leu2d-12057	ACC CTC ACT AAA GGG CGG CCG CAA CCA TGG AGA TAA TGA ATT TCT CTC TCA AC	GTC ATC CTT GTA ATC CAT CGA TAC ATT TCC TGC AAC GGA GAT GCT G	1580
pESC-Leu2d-2384	ACC CTC ACT AAA GGG CGG CCG CAA CCA TGG CGT CTA TCT GTC TCT ACT TTC	GTC ATC CTT GTA ATC CAT CGA TAC AAT CTG AGC AAG TAG ATT GAG C	1536
pESC-Leu2d-1138	ACC CTC ACT AAA GGG CGG CCG CAA CCA CCA TGG AGC TCA TCT TTC TCT TCT	GTC ATC CTT GTA ATC CAT CGA TAC GCG ATC TCT TTT TTC AGA GCA GAA	1503
pESC-Leu2d-3859	ACC CTC ACT AAA GGG CGG CCG CAA CCA CCA TGG CAA TCA AGA TTG C	GTC ATC CTT GTA ATC CAT CGA TAC GCC CAT TTT GCA GAA GAA CAA GCA	1458
pESC-Leu2d-32520	ACC CTC ACT AAA GGG CGG CCG CAA CCA CCT TGT TCA TGA AGT CCA CCC TTG A	GTC ATC CTT GTA ATC CAT CGA TAC GCG CCT GAA GAA TTT CTC CT	1566

References for supporting information

[1] R. Wehrens, L. M. C. Buydens, *J. Stat. Softw.* **2007**, *21*, 1–19.

[2] R. M. E. Payne, D. Xu, E. Foureau, M. Ines, S. Teto, A. Oudin, T. D. De Bernonville, V. Novak, M. Burow, C. Olsen, et al., *Nat. Plants* **2017**, *16208*, 1–9.

[3] D. Ro, M. Ouellet, E. M. Paradise, H. Burd, D. Eng, C. J. Paddon, J. D. Newman, J. D. Keasling, **2008**, *14*, 1–14.

[4] J. J. Maresh, L. A. Giddings, A. Friedrich, E. a. Loris, S. Panjikar, B. L. Trout, J. Stöckigt, B. Peters, S. E. O'Connor, *J. Am. Chem. Soc.* **2008**, *130*, 710–723.

[5] E. Góngora-Castillo, K. L. Childs, G. Fedewa, J. P. Hamilton, D. K. Liscombe, M. Magallanes-Lundback, K. K. Mandadi, E. Nims, W. Runguphan, B. Vaillancourt, et al., *PLoS One* **2012**, *7*, e52506.

[6] H. Schübel, A. Treiber, J. Stökigt, *Helv. Chim. Acta* **1984**, *67*, 2078–2081.

[7] R. Jokela, M. Lounasmaa, *Planta Med* **1996**, 577–579.

[8] C. V. Ferreira Batista, J. Schripsema, R. Verpoorte, S. Beatriz Rech, A. T. Henriques, *Phytochemistry* **1996**, *9422*, 969–973.
